# A validated analysis pipeline for mass spectrometry-based vitreous proteomics: new insights into proliferative diabetic retinopathy

**DOI:** 10.1186/s12014-021-09328-8

**Published:** 2021-12-03

**Authors:** Sarah R. Weber, Yuanjun Zhao, Jingqun Ma, Christopher Gates, Felipe da Veiga Leprevost, Venkatesha Basrur, Alexey I. Nesvizhskii, Thomas W. Gardner, Jeffrey M. Sundstrom

**Affiliations:** 1 Department of Ophthalmology, Penn State College of Medicine, 500 University Drive, Hershey, PA 17033 USA; 2 Kellogg Eye Center, University of Michigan Medical School, 1000 Wall Street, Ann Arbor, MI 48105 USA; 3 Department of Pathology, St. Jude Children’s Research Hospital, 262 Danny Thomas Place, Memphis, TN 38105 USA; 4 Bioinformatics Core, Biomedical Research Core Facilities, University of Michigan Medical School, 2800 Plymouth Road, Ann Arbor, MI 48109 USA; 5 Department of Pathology, University of Michigan Medical School, 1301 Catherine Street, Ann Arbor, MI 48109 USA; 6 Department of Computational Medicine and Bioinformatics, University of Michigan, 100 Washtenaw Ave, Ann Arbor, MI 48109 USA

**Keywords:** Mass spectrometry, Proteomics, Power analysis, Vitreous, Retinal disease, Precision medicine, Diabetic retinopathy

## Abstract

**Background:**

Vitreous is an accessible, information-rich biofluid that has recently been studied as a source of retinal disease-related proteins and pathways. However, the number of samples required to confidently identify perturbed pathways remains unknown. In order to confidently identify these pathways, power analysis must be performed to determine the number of samples required, and sample preparation and analysis must be rigorously defined.

**Methods:**

Control (n = 27) and proliferative diabetic retinopathy (n = 23) vitreous samples were treated as biologically distinct individuals or pooled together and aliquoted into technical replicates. Quantitative mass spectrometry with tandem mass tag labeling was used to identify proteins in individual or pooled control samples to determine technical and biological variability. To determine effect size and perform power analysis, control and proliferative diabetic retinopathy samples were analyzed across four 10-plexes. Pooled samples were used to normalize the data across plexes and generate a single data matrix for downstream analysis.

**Results:**

The total number of unique proteins identified was 1152 in experiment 1, 989 of which were measured in all samples. In experiment 2, 1191 proteins were identified, 727 of which were measured across all samples in all plexes. Data are available via ProteomeXchange with identifier PXD025986. Spearman correlations of protein abundance estimations revealed minimal technical (0.99–1.00) and biological (0.94–0.98) variability. Each plex contained two unique pooled samples: one for normalizing across each 10-plex, and one to internally validate the normalization algorithm. Spearman correlation of the validation pool following normalization was 0.86–0.90. Principal component analysis revealed stratification of samples by disease and not by plex. Subsequent differential expression and pathway analyses demonstrated significant activation of metabolic pathways and inhibition of neuroprotective pathways in proliferative diabetic retinopathy samples relative to controls.

**Conclusions:**

This study demonstrates a feasible, rigorous, and scalable method that can be applied to future proteomic studies of vitreous and identifies previously unrecognized metabolic pathways that advance understanding of diabetic retinopathy.

**Supplementary Information:**

The online version contains supplementary material available at 10.1186/s12014-021-09328-8.

## Background

Over the last two decades, precision medicine methods have revolutionized patient care by leveraging big data to guide medical decision making. For example, cancer researchers regularly interpret and identify actionable information in ‘omic’ datasets to improve understanding of disease heterogeneity, drug target discovery, and predictive markers of drug response and disease progression [1–3]. The use of drugs such as trastuzumab, erlotinib, and crizotinib is the result of -omic data-driven research and has transformed cancer care, permitting data-driven, individualized treatments [4].

A similar large-scale approach is now needed in ophthalmology, where an aging population and unhealthy lifestyles have led to rapidly growing populations of patients with age-related macular degeneration (AMD) and diabetic retinopathy (DR), vision-threatening diseases which now affect more than 100 million patients worldwide [5–7]. Both AMD and DR are heterogeneous in terms of disease onset, progression, and severity and are thus ill-suited for a one-size-fits-all treatment approach.

Currently, intravitreal anti-vascular endothelial growth factor (VEGF) treatments are the mainstay of treatment for late-stage AMD and DR. However, according to recent meta-analyses, only about 1 in 3 patients respond well to this treatment, defined as a gain of 3 or more lines of visual acuity at 1 year [8, 9]. Seven years after treatment, only 23% of patients maintain a best corrected visual acuity of 20/40 or better [10], which is the minimum visual acuity required for legal driving. Phase III clinical trials testing novel, targeted treatments against complement factor D (Roche: lampalizumab) and platelet-derived growth factor (Ophthotech/Novartis: pegpleranib) for these diseases have failed [11, 12], leaving ophthalmologists with only a single, inconsistently effective drug target option. Importantly, access to companion diagnostic tests to assess up- or down-regulation of the treatment target in individual patients randomized in the trials may have facilitated proper stratification and patient selection in these failed trials. The currently limited treatment approach and recent clinical trial failures highlight the importance of using biomarkers for patient stratification and targeted therapy, as is standard care in oncology. Unfortunately, the absence of usable ocular data continues to restrict such efforts.

Effective application of precision medicine to ophthalmology has three requirements. First, a relevant tissue must be acquired from patients with the disease of interest. In the case of retinal diseases like AMD and DR, tissue access is limited due to the inability to obtain retinal tissue from living patients for obvious ethical reasons. However, our group and others have established that vitreous can serve as an information-rich proximal biofluid of the retina to identify proteins and pathways altered in retinal disease [13–17]. Further, we have shown that vitreous fluid can be safely and easily biopsied in a clinical setting [18]. Therefore, vitreous is an ideal tissue to meet this precision medicine requirement. The second requirement for precision medicine is molecular profiling of the obtained tissue. A popular profiling method is shotgun proteomics, as it is unbiased, yields large datasets, and permits analysis at the protein level, the level at which the majority of cellular functions are carried out. Following molecular analysis, a third requirement for precision medicine is the development of a specific therapeutic agent targeting one of its molecular components, presumably one that is dysregulated in patients with the disease of interest.

To fulfill these requirements and begin to develop a precision medicine approach to retinal disease, we apply vitreous proteomics in the same way that cancer researchers have utilized -omic datasets for decades: to understand disease variability between individuals, identify salient molecular profiles, and nominate prognostic and predictive biomarkers. As a first step in this endeavor, the current study describes a feasible, rigorous, and scalable method for proteomic studies of vitreous, specifically focusing on assessing biological and technical reproducibility and the minimum number of samples required to generate statistically significant results when comparing across disease groups. The second step involves analysis of the signaling pathways that are up- and downregulated in vitreous from patients with proliferative diabetic retinopathy (PDR). Moreover, the considerations of statistical power in this study emphasize the necessity of larger sample sizes and the consequent value of sample multiplexing via isobaric tagging as well as normalization across multiple instrument runs. These approaches reveal previously unrecognized metabolic pathways in vitreous of persons with PDR.

## Methods

### Vitrectomy samples

This study was approved by the University of Michigan Institutional Review Board (IRB 00052709) and adhered to the tenets of the Declaration of Helsinki. The vitreous samples were collected in the operating room before clinically indicated vitrectomy as part of a larger protocol establishing a vitreous biorepository at the University of Michigan. Control samples were derived from patients with a vitreoretinal condition resulting from vitreous detachment, macular hole (MH), or epiretinal membrane (ERM). These conditions represent anatomical lesions of an otherwise healthy retina and therefore serve as acceptable controls [13, 19, 20]. Disease samples were obtained from patients with PDR complicated by non-clearing vitreous hemorrhage. Patients were confirmed to have no history of the following ocular conditions: branch retinal vein occlusion within one year of sample collection, age-related macular degeneration, diabetic retinopathy (except patients in the disease group), glaucoma, and uveitis. Patients with a history of cancer (excluding basal or squamous cell carcinoma) and/or diabetes (except in the disease group) were excluded from the study due to potential effects of these systemic diseases on the vitreous proteome that could confound results (Tables 1, 2). Sequential samples obtained between 9/11/14 and 9/28/18 were analyzed as part of the current study; samples meeting the inclusion/exclusion criteria were selected consecutively in the order they were collected from the operating room. Briefly, the procedure was performed in the operating room and either general or local anesthesia was induced. Trocars were used to place three cannulas in the usual fashion, and, with the infusion off, the vitrector was placed into the mid-vitreous cavity. The surgical assistant applied gentle aspiration to an attached 3 mL syringe, and ~0.25 mL vitreous fluid was collected. The syringe was placed on wet ice and immediately placed in a − 80 °C freezer adjacent to the operating room.

**Table 1:** Experiment 1 vitrectomy patient demographics

Plex	Study ID	Exp 1: analyzed individually or pooled	Experiment phenotype	Surgical indication	Sex	Age	Lens status	Past ocular history		
								PVD	Cataract	Other
1.1	203	Treated individually	CTL	ERM OS	M	69	Phakic	Yes	Yes	None
1.1	210	Treated individually	CTL	ERM OD	F	67	Phakic	Yes	Yes	None
1.1	298	Treated individually	CTL	ERM OD	M	73	Pseudophakic	Yes	Yes	None
1.1	322	Treated individually	CTL	ERM OD	F	66	Phakic	Yes	Yes	None
1.1	415	Treated individually	CTL	ERM OD	M	76	Pseudophakic	Yes	Yes	None
1.1	200	Pooled	CTL	ERM OD	M	78	Pseudophakic	Yes	Yes	None
1.1	254	Pooled	CTL	ERM OS	M	80	Phakic	Yes	Yes	None
1.1	278	Pooled	CTL	ERM OS	F	76	Pseudophakic	Yes	Yes	None
1.1	289	Pooled	CTL	ERM OD	F	59	Pseudophakic	Yes	Yes	None
1.1	297	Pooled	CTL	ERM OD	M	86	Pseudophakic	Yes	Yes	None
1.1	300	Pooled	CTL	ERM OD	F	58	Phakic	Yes	Yes	Optic disc drusen associated with ERM
1.1	330	Pooled	CTL	ERM OD	F	70	Phakic	Yes	Yes	Intraretinal cyst, pseudohole
1.1	342	Pooled	CTL	ERM OS	F	62	Pseudophakic	Yes	Yes	None
1.1	424	Pooled	CTL	ERM OS	F	50	Phakic	Yes	No	None
1.1	456	Pooled	CTL	ERM OD	F	67	Pseudophakic	Yes	Yes	None

Clinical information for patients from whom individual control samples and Pool1 aliquots were obtained. *ERM* epiretinal membrane, *OS* oculus sinister (left eye), *OD* oculus dexter (right eye), *PVD* posterior vitreous detachment

**Table 2:** Experiment 2 vitrectomy patient demographics

Plex	Study ID	Subphenotype	Exp 2: used in pool 2	Surgical indication	Sex	Age	Lens status	Past ocular history			Intravitreal anti-VEGF prior to surgery	Weeks between last injection and sample collection	Prior PRP	Weeks between last laser and sample collection	Hemoglobin Conc. (g/dL)	Bilirubin Conc. (mg/dL)
								PVD	Cataract	Other						
2.1	193	CTL	Yes	MH/VMT OS	F	62	Pseudophakic	Yes	Yes	None	No	–	No	–	–	–
2.1	229	CTL	Yes	ERM/MH OD	M	71	Phakic	Yes	Yes	None	No	–	No	–	–	–
2.1	232	CTL	No	ERM/MH OD	F	49	Phakic	Yes	Yes	None	No	–	No	–	–	–
2.1	423	PDR-L	No	PDR: NCVH OD	M	49	Phakic	Yes	Yes	None	Yes	4	Yes	Unknown	0	0.00225
2.1	503	PDR-L	Yes	PDR: NCVH OS	F	71	Pseudophakic	Yes	Yes	ERM	No	–	Yes	13	0	0.00235
2.1	508	PDR-L	No	PDR: NCVH OS	M	49	Phakic	Yes	Yes	None	No	–	Yes	11	0.00010	0.00319
2.1	516	PDR-M	Yes	PDR: NCVH OD	M	56	Pseudophakic	Yes	Yes	None	Yes	2	Yes	Unknown	0.00050	0.00262
2.1	526	PDR-M	Yes	PDR: NCVH OS	F	27	Phakic	Yes	No	None	Yes	6	Yes	7	0.00020	0.00271
2.1	–	Pool 1.6														
2.1	–	Pool 2.1														
2.2	242	CTL	Yes	ERM/MH OD	F	68	Phakic	Yes	Yes	None	No	–	No	–	–	–
2.2	334	CTL	No	ERM OS	M	77	Phakic	Yes	Yes	Hyperopia	No	–	No	–	–	–
2.2	377	CTL	Yes	MH OS	F	59	Phakic	Yes	Yes	None	No	–	No	–	–	–
2.2	554	PDR-L	Yes	PDR: NCVH OS	M	37	Phakic	Yes	Yes	None	No	–	Yes	63	0	0.00231
2.2	715	PDR-L	No	PDR: NCVH OS	M	42	Phakic	No	Yes	None	Yes	33	Yes	40	0	0.00205
2.2	728	PDR-L	Yes	PDR: NCVH OD	F	34	Phakic	No	Yes	None	Yes	1	Yes	24	0	0.00332
2.2	661	PDR-M	Yes	PDR: NCVH OS	F	64	Pseudophakic	Yes	Yes	None	Yes	20	Yes	Unknown	0.00080	0.00281
2.2	808	PDR-M	Yes	PDR: NCVH OD	F	56	Pseudophakic	Yes	Yes	None	Yes	6	Yes	Unknown	0.00030	0.00912
2.2	–	Pool 1.7														
2.2	–	Pool 2.2														
2.3	519	CTL	Yes	MH OS	F	76	Pseudophakic	Yes	Yes	None	No	–	No	–	–	–
2.3	546	CTL	Yes	MH OD	F	74	Phakic	Yes	Yes	None	No	–	No	–	–	–
2.3	780	PDR-L	Yes	PDR: NCVH OD	M	53	Phakic	Yes	Yes	None	Yes	62	Yes	Unknown	0	0.00276
2.3	842	PDR-L	Yes	PDR:NCVH OD	F	58	Phakic	Yes	Yes	None	Yes	2	Yes	Unknown	0	0.00252
2.3	856	PDR-L	Yes	PDR: NCVH OS	F	55	Phakic	Yes	Yes	ERM	Yes	30	Yes	Unknown	0	0.00247
2.3	942	PDR-L	Yes	PDR: NCVH OD	F	45	Phakic	Yes	Yes	ERM	No	–	Yes	13	0	0.00246
2.3	890	PDR-M	Yes	PDR: NCVH OS	F	73	Pseudophakic	Yes	Yes	None	Yes	5	Yes	Unknown	0.00170	0.00280
2.3	934	PDR-M	Yes	PDR: NCVH OD	M	52	Phakic	Yes	Yes	None	No	–	Yes	Unknown	0.00140	0.00244
2.3	-	Pool 1.8														
2.3	-	Pool 2.3														
2.4	875	CTL	Yes	MH OS	F	69	Pseudophakic	Yes	Yes	None	No	–	No	–	–	–
2.4	988	CTL	Yes	MH OD	F	56	Phakic	Yes	Yes	None	No	–	No	–	–	–
2.4	315	PDR-H	No	PDR: NCVH OS	M	54	Phakic	Yes	Yes	None	No	–	Yes	Unknown	0.00660	0.00366
2.4	414	PDR-H	No	PDR: NCVH OD	F	45	Phakic	Yes	Yes	None	Yes	33	Yes	108	0.00520	0.00317
2.4	449	PDR-H	No	PDR: NCVH OS	M	54	Phakic	Yes	Yes	None	Yes	26	Yes	Unknown	0.00840	0.00286
2.4	523	PDR-H	No	PDR: NCVH OS	M	44	Phakic	Yes	No	None	No	–	Yes	Unknown	0.00510	0.00327
2.4	632	PDR-H	No	PDR: NCVH OS	F	78	Pseudophakic	Yes	Yes	None	No	–	Yes	Unknown	0.00490	0.00342
2.4	682	PDR-H	No	PDR: NCVH OS	F	58	Phakic	Yes	Yes	None	Yes	10	Yes	Unknown	0.00500	0.00316
2.4	–	Pool 1.9														
2.4	–	Pool 2.4														
–	951	PDR-L	Yes	PDR: NCVH OD	M	47	Phakic	N	Yes		N	–	Yes	6	0.00000	0.00245

Clinical information for patients from whom individual control and PDR samples and Pool2 aliquots were obtained

*OS* oculus sinister (left eye), *OD* oculus dexter (right eye), *MH/VMT* macular hole/vitreomacular traction, *ERM* epiretinal membrane, *PDR: NCVH* proliferative diabetic retinopathy: non-clearing vitreous haemorrhage, *PVD* posterior vitreous detachment, *VEGF* vascular endothelial growth factor, *PRP* pan-retinal photocoagulation

### Experimental design

**Fig. 1:**
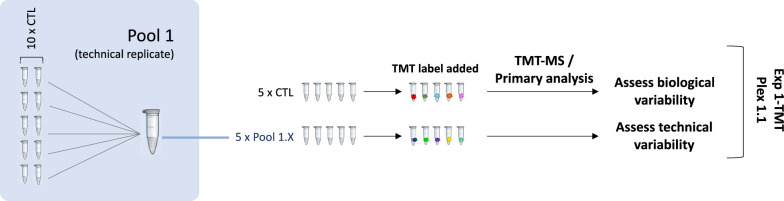
Experiment 1 design. A single 10-plex (1.1) containing individual control samples and pool 1 aliquots was subjected to TMT-MS. The 5 individual control samples served as biological replicates, while the pool 1 aliquots (1.1–1.5) served as technical replicates

**Fig. 2:**
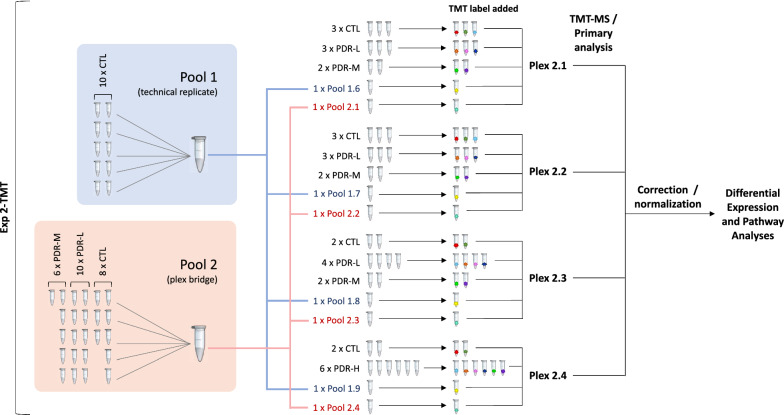
Experiment 2 design. Four 10-plexes (2.1–2.4), each containing a combination of control, PDR, and pool samples, were subjected to TMT-MS on the same day. Pool 1 aliquots were made from the same pool of control samples used in experiment 1 and served as technical replicates. Pool 2 aliquots were derived from a mixture of control and PDR samples in order to reflect the complexity of individual samples used in experiment 2. Data from plexes 2.1–2.4 were then aggregated into a normalized expression matrix. Matrix data were used for statistical and bioinformatic analyses

The following experiments compare a disease group (PDR samples) to a control group (MH/ERM samples); the characteristics of these groups are detailed in the prior section. Patient demographics according to their distribution across experiments are shown in Tables 1 and 2. Schematic representations of the experimental design are provided in Figs. 1 and 2. For the purpose of this study, the term “experiment” represents a set of samples analyzed on the same day according to the aforementioned schematic, whereas a “plex” refers to a set of samples run simultaneously as part of a labeled kit.

Experiment 1 (Fig. 1) subjected a single 10-plex (plex 1.1) to tandem mass tag-mass spectrometry (TMT-MS). The 10-plex consisted of five control (MH/ERM) samples derived from five different patients to serve as biological replicates. The remaining five samples consisted of aliquots of a pooled mixture of control samples (pools 1.1–1.5) from 10 different patients. These five pool aliquots can be assumed to have identical compositions, so they served as technical replicates.

Experiment 2 (Fig. 2) subjected four 10-plexes (plexes 2.1–2.4) containing both control (MH/ERM) and disease (PDR) samples derived from individual patients. PDR samples were subdivided into low (PDR-L), medium (PDR-M), and high (PDR-H) subphenotype groups based on their relative hemoglobin concentrations to assess whether this parameter reflects an alteration in the vitreous proteome. Aliquoted samples from two pools were also distributed across each plex. Pools 1.6–1.9 derived from the initial control sample pool created in experiment 1 and, as in experiment 1, served as technical replicates. Pools 2.1–2.4 derived from an aggregated mixture of both control and PDR samples (pool 2) and served as a plex bridge, permitting assessment of plex-to-plex variability for samples run on a given day. Both control and disease samples were used for pool 2 in an effort to mirror the compositional complexity of the samples analyzed in experiment 2. Data from all 4 plexes from experiment 2 were aggregated into a single, normalized expression matrix. Matrix data were used to perform a power analysis and to create plots showing technical variability within and across plexes. Differential expression analysis was performed to identify molecular profiles underlying proteome differences between control, disease, and disease subphenotype groups. This analysis utilized literature-defined gene sets to determine associations with biological mechanisms and disease processes.

### Proteomics

#### Sample processing

**Fig. 3:**
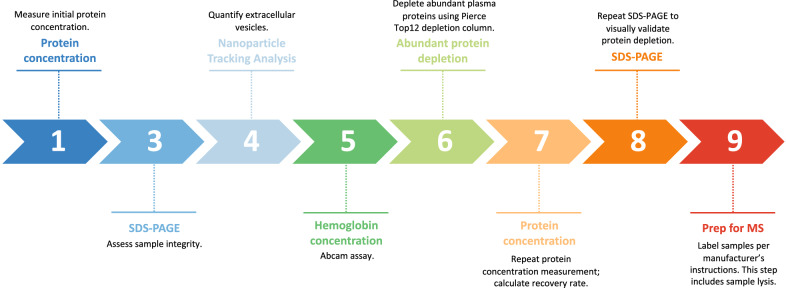
Sample processing workflow. Sequential sample validation and processing steps are outlined here

Prior to MS analysis, samples were processed as was done previously [14], with minor changes. An overview of this process is shown in Fig. 3. Briefly, vitreous samples were thawed on ice and spun at 17,000 g for 30 min at 4 °C to remove large cellular debris such as collagen fibers and glycosaminoglycans. Of note, prior studies have shown that extracellular vesicles (EVs) remain buoyant at this speed [14, 21]. Supernatant was transferred to new tube. Each sample was run on SDS-PAGE to assess its integrity. Hemoglobin and bilirubin concentrations were measured via assays (AbCam, Cambridge, UK) in all PDR samples to assess whether these factors contributed to the variably tinted gross appearance of a subgroup of samples. Total protein concentration was measured via DC Protein Assay Reagents (5000116, Bio-Rad, Hercules, CA, USA) before and after abundant protein depletion (Additional file 1). Samples for protein assay were prepared as follows: 2.5 μL protein sample, 0.5 μL 10 × RIPA buffer (9806, Cell Signaling Technology, Danvers, MA, USA), and 2 μL H_2_O were mixed and incubated on ice for 30 min. Abundant proteins were depleted using a Pierce™ Top 12 protein depletion spin column (85165, Thermo Fisher, Waltham, MA, USA; depletes α1-acid glycoprotein, α1-antitrypsin, α2-macroglobulin, albumin, apolipoprotein A-I, apolipoprotein A-II, fibrinogen, haptoglobin, IgA, IgG, IgM, and transferrin) to avoid masking proteins present in lower amounts; 250 µg of protein were loaded onto the column and incubated with gentle end-over-end mixing for 2 h at RT. Filtrate and wash fractions were combined and concentrated to ~ 40 µL using Amicon Ultra-0.5 Centrifugal Filter Device (NMWL 3 K, UFC500396, MilliporeSigma, Burlington, MA, USA) by spinning at 14,000 g at 4 °C. The depleted and concentrated vitreous was recovered by spinning the column upside down at 1000 g for 2 min at 4 °C. The samples were snap frozen in liquid nitrogen and stored at − 80 °C.

Because PDR samples had differing hues of red, yellow, or colorless on gross inspection, samples were classified into subphenotype groups according to relative low, medium, or high hemoglobin concentration. PDR-L samples were those that contained ≤ 1.00 × 10^–4^ g/dL hemoglobin. PDR-M samples had hemoglobin concentrations between 2.00 × 10^–4^ and 1.70 × 10^–3^ g/dL, and PDR-H samples had hemoglobin concentrations between 4.90 × 10^–3^ and 8.40 × 10^–3^ g/dL. For reference, the concentration of hemoglobin in human blood is ~15 g/dL; therefore, even the highest concentration of hemoglobin measured in vitreous samples in this study is 1785 times lower than that of blood, or 0.056%. Vitreous samples containing ~500 μg protein or greater were analyzed individually. Pool 1 was created by combining the full volumes of the control samples indicated in Table 1. Pool 2 was created by combining 5 µg protein from the control and PDR samples indicated in Table 2. These mixtures were then aliquoted into smaller volumes containing 20 µg protein each (pools 1.X and 2.X). Samples were distributed across two experiments consisting of five 10-plexes total according to the schematics laid out in Figs. 1 and 2. The transproteomic pipeline was applied to the dataset in accordance with the current Human Proteome Organization guidelines.

#### Protein digestion and TMT-labeling

Samples were labeled using a TMT 10-plex kit (ThermoFisher Scientific) using methods described by Tank et al. [22]. Briefly, 20 µg depleted and concentrated vitreous was mixed with 5 µL 10 × RIPA buffer and DPBS to final volume of 50 µL, then incubated on ice for 30 min. For pooled sample, 2.5 µg vitreous from each individual sample (32 total) was taken, mixed with 20 µL 10 × RIPA buffer and DPBS to a final volume of 200 µL, then incubated on ice for 30 min and aliquoted into 4 tubes (20 µg protein in 50 µL each).

Samples (20 µg/sample) were proteolysed and labeled with TMT 10-plex essentially by following manufacturer’s protocol (ThermoFisher). Briefly, upon reduction (5 mM DTT, for 30 min at 45 °C) and alkylation (15 mM 2-chloroacetamide, for 30 min at room temperature) of cysteines, the proteins were precipitated by adding 6 volumes of ice-cold acetone followed by overnight incubation at − 20° C. The precipitate was spun down, and the pellet was allowed to air dry. The pellet was resuspended in 0.1 M TEAB, and overnight (~ 16 h) digestion with trypsin/Lys-C mix (1:25 protease:protein; Promega) at 37 °C was performed with constant mixing using a thermomixer. The TMT 10-plex reagents were dissolved in 41 µL of anhydrous acetonitrile and labeling was performed by transferring the entire digest to TMT reagent vial and incubating at room temperature for 1 h. Reaction was quenched by adding 8 µL of 5% hydroxyl amine and further 15 min incubation. Labeled samples were mixed together and dried using a vacufuge. An offline fractionation of the combined sample (~ 200 µg) into 8 fractions was performed using high pH reversed-phase peptide fractionation kit according to the manufacturer’s protocol (Pierce; Cat #84868). Fractions were dried and reconstituted in 9 µL of 0.1% formic acid/2% acetonitrile in preparation for LC–MS/MS analysis.

#### Liquid chromatography–mass spectrometry analysis (LC-multinotch MS3)

To obtain superior quantitative accuracy, we employed multinotch-MS3 (McAlister GC), as described by McAlister et al. [23]. This technique minimizes the reporter ion ratio distortion resulting from fragmentation of co-isolated peptides during MS analysis. Orbitrap Fusion (Thermo Fisher Scientific) and RSLC Ultimate 3000 nano-UPLC (Dionex) were used to acquire the data. Two μL of the sample was resolved on a PepMap RSLC C18 column (75 μm i.d. × 50 cm; Thermo Scientific) at the flow-rate of 300 nL/min using 0.1% formic acid/acetonitrile gradient system (2–22% acetonitrile in 150 min; 22–32% acetonitrile in 40 min; 20 min wash at 90% followed by 50 min re-equilibration) and directly sprayed onto the mass spectrometer using EasySpray source (Thermo Fisher Scientific). The mass spectrometer was set to collect one MS1 scan (Orbitrap; 120 K resolution; AGC target 2 × 105; max IT 100 ms) followed by data-dependent, “Top Speed” (3 s) MS2 scans (collision induced dissociation; ion trap; NCE 35; AGC 5 × 103; max IT 100 ms). For multinotch-MS3, top 10 precursors from each MS2 were fragmented by HCD followed by Orbitrap analysis (NCE 55; 60 K resolution; AGC 5 × 104; max IT 120 ms, 100–500 *m*/*z* scan range).

#### Initial mass spectrometry data processing

Raw mass spectrometry files were converted into open mzML format using msconvert utility of Proteowizard software suite. MS/MS spectra were searched using the MSFragger database search tool (Kong et al. 2017) against a Uniprot—SwissProt protein sequence database, appended with an equal number of decoy sequences, downloaded on February 02, 2020. MS/MS spectra were searched using a precursor-ion mass tolerance of 20 p.p.m., fragment mass tolerance of 0.6 Da, and allowing C12/C13 isotope errors (− 1/0/1/2/3). Cysteine carbamylation (+ 57.0215) and lysine TMT labeling (+ 229.1629) were specified as fixed modifications, and methionine oxidation (+ 15.9949), N-terminal protein acetylation (+ 42.0106), and TMT labeling of peptide N-terminus and serine residues were specified as variable modifications. The search was restricted to fully tryptic peptides, allowing up to two missed cleavage sites. The search results were further processed using the Philosopher pipeline [24]. First, MSFragger output files (in pepXML format) were processed using PeptideProphet [25] (with the high-mass accuracy binning and semi-parametric mixture modeling options) to compute the posterior probability of correct identification for each peptide to spectrum match (PSM). The resulting pepXML files from PeptideProphet (or PTMProphet) from all 23 TMT 10-plex experiments were then processed together to assemble peptides into proteins (protein inference) and to create a combined file (in protXML format) of high confidence proteins groups and the corresponding peptides assigned to each group. The combined protXML file, and the individual PSM lists for each TMT 10-plex, were further processed using the Philosopher filter command. Each peptide was assigned either as a unique peptide to a particular protein group or assigned as a razor peptide to a single protein group with the most peptide evidence. The protein groups assembled by ProteinProphet [26] were filtered to 1% protein-level False Discovery Rate (FDR) using the chosen FDR target-decoy strategy and the best peptide approach (allowing both unique and razor peptides) and applying the picked FDR strategy [27]. In each TMT 10-plex, the PSM lists were filtered using a stringent, sequential FDR strategy keeping only PSMs with PeptideProphet probability of 0.9 or higher (which in these data corresponded to less than 1% PSM-level FDR) and mapped to proteins that also passed the global 1% protein-level FDR filter. For each PSM passing these filters, MS1 intensity of the corresponding precursor-ion was extracted using the Philosopher label-free quantification module based on the moFF method [28] (using 20 p.p.m mass tolerance and 0.4 min retention time window for extracted ion chromatogram peak tracing). For all PSMs corresponding to a TMT-labeled peptide, ten TMT reporter ion intensities were extracted from the MS/MS scans (using a 0.002 Da window). The precursor ion purity scores were calculated using the sequenced precursor ion’s intensity and other interfering ions observed in MS1 data (within a 0.7 Da isolation window). All supporting information for each PSM, including the accession numbers and names of the protein/gene selected based on the protein inference approach with razor peptide assignment, and quantification information (MS1 precursor-ion intensity and the TMT reporter ion intensities) were summarized in the output PSM tables.

#### Normalization across TMT plexes

The PSM tables from above were further processed using TMT-Integrator (https://github.com/Nesvilab/TMT-Integrator) to generate the gene’s summary reports and protein level. In the quantification step, TMT- Integrator used as input the PSM tables generated by the Philosopher pipeline as described above and created integrated reports with quantification across all samples at each level. Each PSM was filtered to remove all entries that did not pass at least one of the quality filters, such as PSMs with (a) no TMT label; (b) missing quantification in the reference sample; (c) precursor-ion purity less than 50%; (d) summed reporter ion intensity (across all ten channels) in the 5th percentile or lower of all PSMs in the corresponding PSM table. In the case of redundant PSMs (i.e., multiple PSMs in the same MS run sample corresponding to the same peptide ion), only the one having the highest summed TMT intensity was kept for subsequent analysis. Both unique and razor peptides were used for quantification, while PSMs mapping to common external contaminant proteins (included in the searched protein sequence database) were excluded. Next, in each TMT experiment, for each PSM, the intensity in each TMT channel was log2 transformed. The reference channel intensity (pooled reference sample) was subtracted from that for the other nine channels (samples), thus converting the data into a log2-based ratio to the reference scale (referred to as ‘ratios’ below). The PSMs were grouped based on a predefined level (gene, protein, and peptide and site-level for phosphopeptide enriched data; see below for details) after the reference conversion ratio. At each level and in each sample, the interquartile range (IQR) algorithm was applied to remove the corresponding PSM group’s outliers. The first quantile (Q1), the third quantile (Q3), and the IQR (i.e., Q3–Q1) of the sample ratios were calculated, and the PSMs with ratios outside of the boundaries of Q1 − 1.5 × IQR and Q3 + 1.5 × IQR were excluded. The median was then calculated from the remaining ratios to represent each sample’s ratio at every level. In the next step, the ratios were normalized using the median absolute deviation (MAD). Briefly, independently at each level of data summarization (gene, protein, peptide, or site), given the p by n table of ratios for entry j in sample i, R ij, the median ratio M i = median(R ij, j = 1,…,p), and the global median across all n samples, M 0 = median(M i, i = 1,…,n), were calculated. The ratios in each sample were median centered, R C ij = R ij – M i. The median absolute deviation of centered values in each sample was calculated, MAD i = median(abs(R C ij), j = 1…p), along with the global absolute deviation, MAD 0 = median(MAD i, i = 1,…,n). All ratios were then scaled to derive the final normalized measures: R N ij = (R C ij/MAD i) × MAD 0 + M 0. As the last step, the normalized ratios were converted back to the absolute intensity scale using each entry’s estimated intensity (at each level, gene/protein/peptide/site) in the Reference sample. The Reference Intensity of entry i measured in TMT 10-plex k (k = 1,…,q), REF ik, was estimated using the weighted sum of the MS1 intensities of the top 3 most intense peptide ions [29] quantified for that entry in the TMT 10-plex k. For each PSM, the weighting factor is taken as the proportion of the reference channel TMT intensity to the total summed TMT channel intensity. The overall Reference Intensity for entry i was then computed as REF i = Mean(REF ik, k = 1,…,q). In doing so, the missing intensity values (i.e., no identified and/or quantified PSMs in a particular TMT 10-plex experiment) were imputed with a global minimum intensity value. The final abundance (intensity) of entry i in sample j (log2 transformed) was computed as A ij = R N ij + log2(REF i). The ratio and intensity tables described above were calculated separately for each level (gene and protein for the whole proteome, and peptide). A normalized gene-level abundance matrix was constructed by grouping all PSMs by the gene symbol of the corresponding protein, assigned as either unique or razor peptides. In the protein tables, identified proteins that mapped to the same gene were kept as separate entries.

#### Differential expression analysis

The full normalized gene-level abundance matrix was used to assess technical and biological variability using pairwise Spearman correlation. Unless noted, analyses of normalized abundance data were based on the log2-based ratio of sample intensity/reference intensity as calculated above. Principal component analysis was performed to assess patterns in variance across sample phenotypes following normalization. To execute differential protein expression analysis, the 36-sample unified normalized matrix was trimmed to remove the 4 pooled samples and also any protein not measured across all samples. Differential analysis was performed using moderated t-statistics from the empirical Bayes procedure linear model for microarray analysis (LIMMA) [30] as extended to accommodate TMT proteomic data [31]. Prospective power analysis plots were modeled using Hedges’ g effect size and the R libraries effsize ssize.fdr [32, 33]. Gene set enrichment and pathway analyses were completed using iPathway Guide (Advaita Corporation, Ann Arbor, MI, USA) and Ingenuity Pathway Analysis (Qiagen Sciences Inc, Germantown, MD, USA). The mass spectrometry proteomics data have been deposited to the ProteomeXchange Consortium via the PRIDE [34] partner repository with the dataset identifier PXD025986. The complete dataset including the complete list of proteins and peptides identified can be found in Additional file 1.

### Analysis of extracellular vesicle size distributions

To visualize and quantify the EV content of unfractionated vitreous samples, Nanoparticle Tracking Analysis (NTA) was performed as described previously [14], where tracked particles are presumed to represent EVs based on prior analysis [14]. This step was performed prior to abundant protein depletion. Briefly, vitreous samples were removed from storage at − 80 °C, thawed on ice, and centrifuged at 2000 g for 30 min at 4 °C. The supernatants were diluted to 1 ml [1:50 to 1:1000] with particle-free water. Each prepared sample was loaded by syringe pump into the NanoSight NS300 (Malvern Instruments Ltd, Malvern, Worcestershire, UK) set to scatter mode, and five 60-s videos were generated at 24.98 frames per second. The size distribution and concentration of particles were calculated using NanoSight software version 3.2 (Malvern Instruments Ltd, Malvern, Worcestershire, UK). All samples in the current data set were run by the same individual. The raw NTA data were processed using Microsoft Excel (Redmond, WA, USA). Individual tracings from a single vitreous sample were averaged, and the averaged data for all samples within a single phenotype were again averaged to yield a final graph. Particle abundance values of zero were replaced with blank cells to reflect their interpretation as undetected rather than truly zero.

## Results

### Analysis of variability across technical and biological replicates

**Fig. 4:**
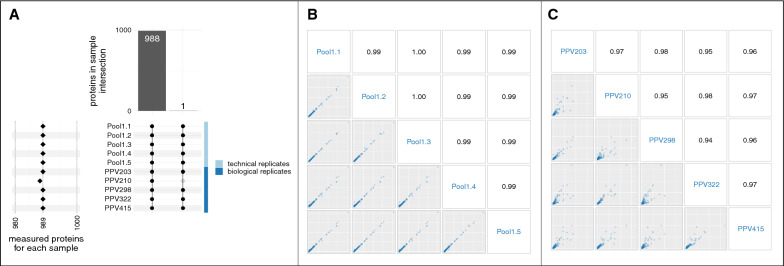
Total proteins confidently identified and quantified within a single TMT plex. **A** An upset plot shows that most detected proteins were assigned abundances in all samples in experiment 1. The lower-left plot shows the number of proteins measured for each sample (row); excepting sample PPV210, all samples quantified abundance for 989 proteins. The center matrix and upper bar plot show how different samples measured the different sets of proteins. Each row represents a sample, and each column represents a set of one or more measured proteins; at the row-column coordinate, a gray node indicates this protein-set was not measured by this sample and a black node indicates this protein-set was measured; black nodes are vertically connected by intersection lines. The first column shows that 988 proteins abundances were assigned across all samples; likewise, 163 proteins were detected but were not assigned an abundance, and one protein was detected in only 9 samples (excluding PPV210). **B** Pairwise scatter plots of normalized protein abundance across pool 1 aliquots run in the same TMT batch in experiment 1 show samples listed in blue down the diagonal and Spearman correlations in black along the upper triangle. The axes show the normalized protein abundance; all axes are identically scaled. **C** Protein abundance as above, but across individual biological replicates. Plots in panels **B** and **C** show nearly perfect Spearman correlation between technical replicates and very strong correlation between biological replicates

Full protein and peptide lists from both experiments can be found in Additional file 1. Experiment 1 (Fig. 1) utilized a 10-plex consisting of 5 individual and 5 pooled sample aliquots to assess biological and technical variability, respectively. Both the overlap of quantified proteins and the distribution of protein abundances across samples were assessed. Of the 1152 detected proteins, 988 were assigned abundances in all samples. One protein, leucine-rich repeats and immunoglobulin-like domains protein 2, was not assigned an abundance in one sample, PPV210 (Fig. 4A). Following normalization, protein abundances across individual biological replicates and technical replicates were assessed via Spearman correlation. A near perfect correlation (0.99–1.00) was observed between technical replicates, and a very strong correlation (0.94–0.98) was seen between biological replicates (Fig. 4B, C). Correlation of abundance estimations across technical and biological replicates validate the sample preparation protocols and proteomic analysis yield consistent results within a single TMT plex.

### Use of normalization to minimize batch effects

Experiment 2 (Fig. 2) utilized four 10-plexes containing 10 control and 22 PDR samples derived from individual patients, 4 Pool 1 aliquots (technical replicates), and 4 Pool 2 aliquots (technical replicates). PDR samples were grouped into 3 categories according to their hemoglobin concentration.

**Fig. 5:**
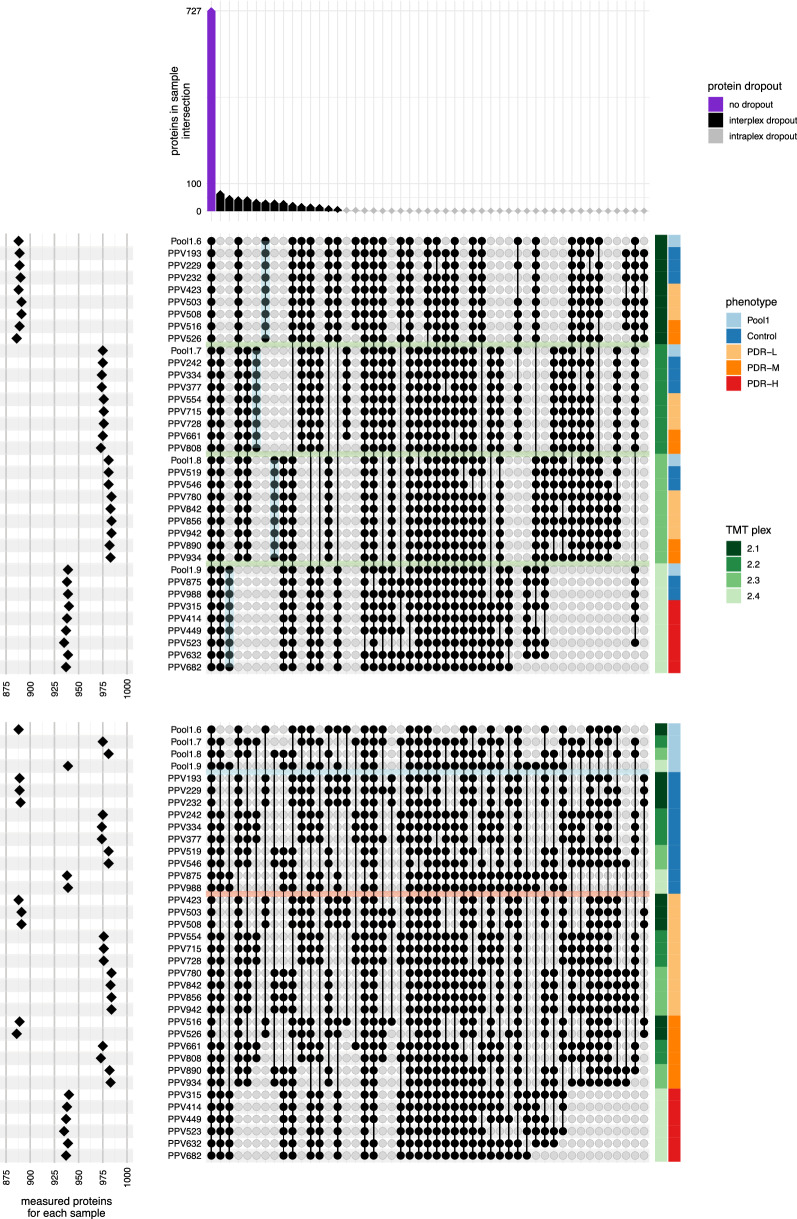
Distribution of identified proteins across all samples and plexes. These two upset plots show that in experiment 2, 1157 distinct proteins were assigned an abundance, but not all proteins were measured in all samples. The two plots represent the same data from different perspectives: the upper plot orders samples by plex, and the lower plot by phenotype. (See Fig. 3 for more details on interpreting the upset figure.) The first column (purple) of the top-most bar plot shows that 727 proteins were measured consistently across all samples. The next 14 columns (black) illustrate inter-plex dropout, where a protein was consistently measured for all samples within one or more plexes but was wholly absent in other plexes. Inter-plex dropout accounted for 390 proteins overall. The remaining 40 columns show intra-plex dropout (gray diamonds), where a protein is not consistently measured within a single plex. In this dataset, intra-plex dropout accounts for 40 proteins. As depicted in the left plot, the number of measured proteins for a given sample correlated strongly with the TMT plex; on average each sample measured 946 proteins, with individual counts ranging from 886 to 984. The horizontal green bands in the upper intersection matrix mark the divisions between plexes. The vertical cyan lines highlight 151 proteins measured by a single plex. In the bottom matrix, the cyan horizontal band across the intersection matrix marks the divide between pooled and individual samples. The intersection lines connecting the nodes indicate the majority of proteins measured in the individual samples were also measured in the pool aliquots and also that all proteins measured by at least one control sample were also measured by at least one PDR sample

**Fig. 6:**
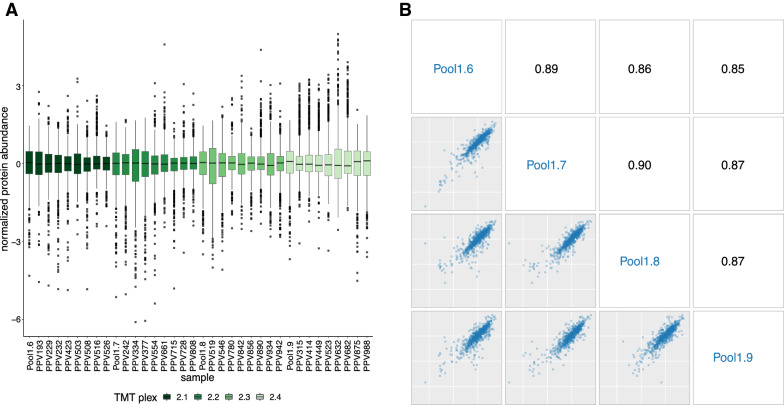
Assessment of protein normalization across plexes. **A** Each boxplot represents the distribution of Z-score normalized expression intensity for all proteins in a sample (see “Methods” for details on how abundances were normalized across plexes). The normalization shows some sample-to-sample variability but produces consistent distributions across all samples across all four plexes, showing no obvious plex bias. **B** In addition to the bridge-channel pool aliquot, each of the four plexes in experiment 2 contained an aliquot of the pooled control samples (pool 1) from experiment 1 as a technical replicate. The pairwise scatter plots of normalized protein intensity across pool 1 aliquots and their corresponding Spearman correlations show excellent correlation across plexes

Of the 1191 proteins assigned an abundance, not all proteins were measured by all samples (Fig. 5). The data dependent MS method used in this study inevitably introduces dropout between TMT plexes due to under sampling. A total of 727 proteins were measured consistently across all samples; 390 proteins were consistently measured by some TMT plexes but not detected in other plexes (interplex dropout); 40 proteins were measured in a subset of samples in a plex (intraplex dropout). The observed interplex dropout rate is in line with similar studies [35]. All 1191 measured proteins were detected in at least one control and one PDR sample. Normalized protein expression showed no obvious plex-bias (Additional file 1). High pairwise Spearman correlations (0.85–0.90) among pools 1.6–1.9 (additional aliquots derived from the same pooled sample mixture used in experiment 1 that were distributed across the 4 plexes in experiment 2) confirm that normalization diminished TMT-plex batch effects. Slight sample variability is apparent, but no obvious plex bias can be discerned (Fig. 6).

### Effect size, power analysis, and their utility in experimental design

This study was an untargeted exploration of differentially expressed proteins in human vitreous. Differential expression was measured on each protein by comparing the distributions of protein abundance between control and PDR samples assuming an H_0_ that the measurements came from the same distribution and H_A_ that the distributions were distinct. See methods above for more details on differential expression analysis. Considering the subset of proteins that were measured across all samples (727) and adjusting for multiple hypothesis testing, 62% had Storey adjusted p-values less than 0.05, and 42% had adjusted p-values less than 0.01 [36, 37] (Additional file 1).

A p-value quantifies confidence that there is a statistically significant difference between groups. A useful and complementary perspective on the data considers the estimated effect sizes between groups [38, 39]. The fold-change measure reported in the differential expression analysis is an absolute effect size which quantifies the difference between the means of the two groups (PDR vs. control); in the context of a specific protein, the absolute effect size between the groups directly relates to the biological mechanism in question, and the effect sizes can help elucidate and prioritize potential mechanisms or biomarkers to elaborate in follow-on investigations.

**Fig. 7:**
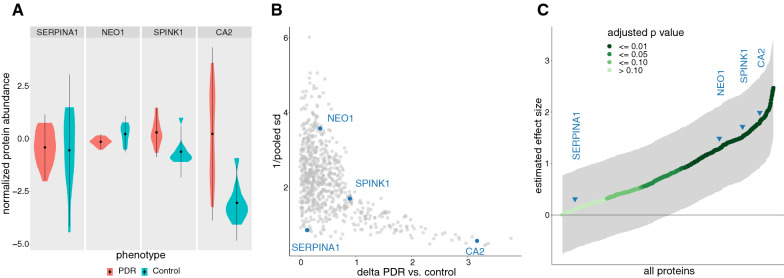
Measurement of effect sizes across select proteins. **A** Distributions of normalized protein abundance. The mean and standard deviation for each distribution is marked by the center dot and line, respectively (note that these proteins were selected to illustrate relevant patterns that impact statistical power). The power for each of these proteins is determined by the overlap of distributions between PDR and control groups. Assuming both groups follow normal distributions, one can compare them quantitatively by considering (a) difference in means (delta PDR vs. control) and (b) a pooled standard deviation that characterizes their dispersions. **B** Scatter plot of protein delta (PDR vs. control) and dispersion. The product of these two coordinates defines the estimate of Hedges’ g relative effect size for each protein (absolute value of the delta considers only the magnitude of the effect). Highlighted proteins illustrate distinct patterns in protein delta and dispersion. **C** Estimated Hedges’ g effect size. The 95% confidence interval of the estimated effect is shaded in gray. As stated above, effect size estimators make specific assumptions about the data. In the Hedges’ g effect estimator, data from each group are assumed to be from a normal distribution where the standard deviations are free from systematic differences. For that reason, effect sizes and power calculations for a specific protein should also include a detailed examination of the actual distributions. Note that the distributions in **A** for protein CA2 appear to violate both these assumptions, so while these distributions appear to be distinct, the calculated effect size for this protein should be treated with skepticism (see Additional file 1). Effect size estimations were performed with the R library effsize (v0.8.1) [125]

The effect size is also a key input in a prospective power analysis, an analysis that predicts the statistical power of a proposed experimental design [40]. The statistical power of a hypothesis test is the probability of correctly rejecting the null hypothesis when there is a true difference between groups; it is calculated by assessing how the two group distributions overlap (in this study, the distributions of protein abundance in control and PDR groups). Key determinants of power include the type of hypothesis test (e.g., t-test), threshold of statistical significance (by convention, alpha is typically set somewhere between 0.01 and 0.10 [41]), the sample sizes of the groups (determined by experimental design and sample availability), and the magnitude of difference between groups. Note that the difference in between groups (PDR vs. control) is an attribute of a specific feature (protein), so each measured feature is assigned a specific power. In a prospective power analysis for a specific feature, to calculate the minimum sample size for a given power, it is necessary to estimate the difference in means (delta) and also the dispersion of the two distributions (the inverse of which represents variance); the difference and dispersion are often combined into a single measure called the relative effect size. Cohen’s d, and Hedges’ g effect sizes are two commonly used and closely related relative effect size measures [42, 43]. The smaller of our two groups (control) has a relatively small sample size (n = 10), so we calculated a relative effect size using Hedges’ g effect size. Proteins chosen for differences in these statistical properties are shown in Fig. 7.

**Fig. 8:**
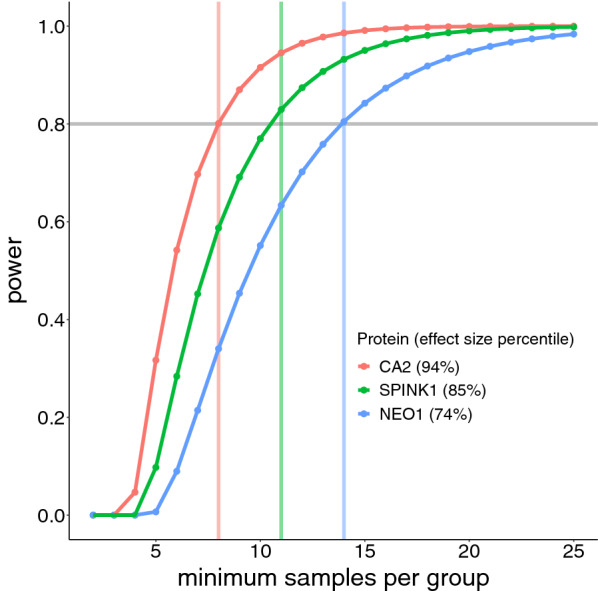
Predicted statistical power of selected proteins plotted against sample size. Given the 10 control samples, the protein SPINK1 (ranked 85th percentile across protein effect sizes) reached a power of just under 0.8 (gray horizontal line). Thus, in future experiments, a protein with similar effect size would correctly identify a statistically significant difference 80% of the time. Note that NEO1 (74th percentile effect size) shows power less than 0.6 at 10 samples and would require 14 samples to reach a power of 0.8. While this experiment combined multiple TMT plexes into a unified abundance matrix, an alternative approach to achieving sufficient sample size could be to use a single 16-plex TMT approach with a balanced experimental design (8 control + 8 test); assuming this simpler, single-plex approach, only those proteins with the highest effect sizes (e.g., protein CA2) could reach a power of 0.8 at 8 samples per group. Note that setting the power threshold to 0.8 is common but arbitrary; in practice, a different threshold may be more appropriate for a given experiment. Note also that CA2 is included for consistency with plots above; while it accurately reflects the stringency of power at lower sample sizes, a precise power calculation for this protein should incorporate the non-normality of dispersions alluded to in previous figures (see Additional file 1). In general, effect size ranges and qualitative measures of effect magnitude (e.g., small, medium, large) can inform the experimental design of untargeted experiments; however, a close examination of abundance distributions in specific proteins of interest enables more meaningful and reliable power calculations. This plot was generated with ssize-fdr R library; the calculations assume an FDR = 0.05, and pi_0_ = 0.7 [33]

Future targeted analyses of specific proteins of interest can leverage the estimated effect sizes (detailed in Additional file 1) to inform the minimum number of samples required for control and test groups; for untargeted experiments, we can also incorporate adjustments for multiple hypothesis testing [37]. Figure 8 shows the relationship of sample size and predicted power for several selected proteins. For example, CA2, which has a high delta and low variance, easily achieves a power of 0.8 at a sample size of 8. SPINK1, which has an intermediate delta and variance, reached a power of just under 0.8 at a sample size of 10. NEO1, which has a low delta and high variance, shows power < 0.6 at a sample size of 10 and would require a sample size of 14 to reach a power of 0.8. In general, while effect size ranges and qualitative measures of effect magnitude (e.g., small, medium, large) can inform the experimental design of untargeted experiments, predicted effect sizes are more meaningful in the context of a specific protein or focused subset of related proteins.

Relatively low statistical power at smaller sample sizes underscores an essential difficulty in structuring an effective untargeted proteomic analysis. At the same time, it accentuates the key advantages of (1) using isobaric labeling to combine distinct samples into a single LCMS run to mitigate technical effects, (2) a consistent/efficient sample preparation protocol to minimize technical variation across samples, (3) a biobank of comprehensively annotated samples to draw from, and (4) combining two or more TMT plexes together using reference channels to normalize estimates of protein abundance. Note that as the number of combined multiplexed runs increases, so too does protein dropout due to proteins not measured in a specific plex. Therefore, when combining multiplexed runs, there is a natural tension between increased sample size and increased protein dropout. Isobaric labeling that accommodates larger numbers of samples per run would ameliorate this limitation. Also, follow-on analyses should consider protein abundance imputation methods to mitigate plex-protein dropout.

### Characterization of disease phenotype

**Fig. 9:**
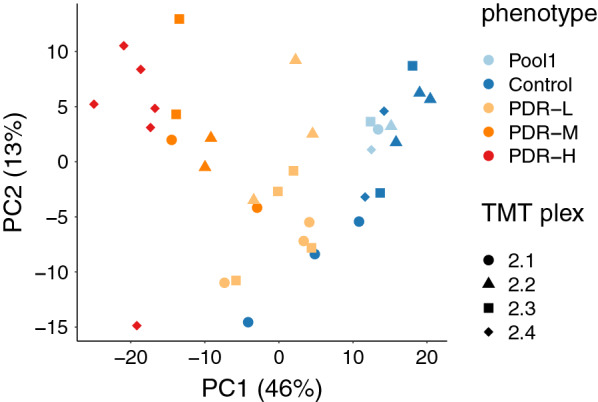
Principal component analysis shows stratification by clinical phenotype. Scatter plot of the samples by first two principal components differentiated by TMT plex (shape) and phenotype (color); component variance noted in parentheses. Note that PCA considered only the subset of proteins measured in all samples

Principal component analysis (PCA) was performed in order to assess the variance across phenotypes, sub-phenotypes, and technical replicates (Fig. 9). PCA shows a good proportion of overall variance explained by the first two components, and also good separation of main phenotypes (control, PDR) and sub-phenotypes (control, PDR-L, PDR-M, PDR-H). The PCA also shows very tight clustering of technical replicates (the pooled controls) and no obvious separation by plex.

**Fig. 10:**
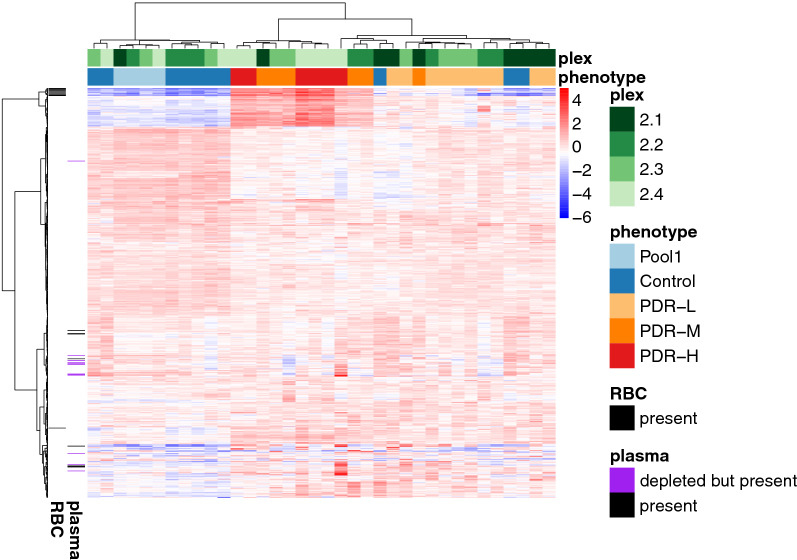
Protein expression heatmap of all consistently measured proteins. Heatmap of normalized protein expression hierarchically clustered by samples and proteins with strip plots of plex, phenotype across samples (columns), and strip plot of plasma protein and red blood cell across proteins (rows). Note that this heatmap and clustering considered only the subset of proteins measured in all samples; hierarchical clustering was based on Euclidean distance of normalized expression using Ward’s method

Hierarchical clustering by samples and proteins was visualized via heatmaps of normalized protein expression, utilizing the subset of proteins that were present in all samples (Fig. 10). Sample clustering showed tight clustering of technical replicates, very good separation between control and PDR groups, and moderate separation of PDR subphenotypes. To test if blood components were affecting analysis, the top 23 abundant plasma proteins, which account for 97% of total plasma protein mass, and 7 proteins that are expressed at levels ≥ 1000-fold higher in erythroid versus non-erythroid cells [44] were annotated. Notably, all 30 plasma and erythrocyte proteins were present in all samples in both PDR and control groups. Abundant plasma proteins were distributed across clusters and showed no enrichments in a specific cluster; erythroid proteins (annotated as red blood cell [RBC]) showed marked enrichment in one cluster. To ensure the plasma and erythroid proteins were not dominating the sample clustering, the heatmap-clustering was rerun excluding those 30 proteins (Additional file 1). This subset recapitulated the tight clustering of technical replicates and separation of control vs. PDR and slightly improved the clustering of PDR subphenotypes. Finally, considering only the subset of plasma and erythroid proteins, the sample clustering was dominated by a single cluster of coexpressing erythroid proteins (Additional file 1). The sample clustering showed reasonably good separation between PDR-H/M, PDR-L, and control subphenotypes (consistent with the subphenotype partitions as assigned by hemoglobin concentration).

**Fig. 11:**
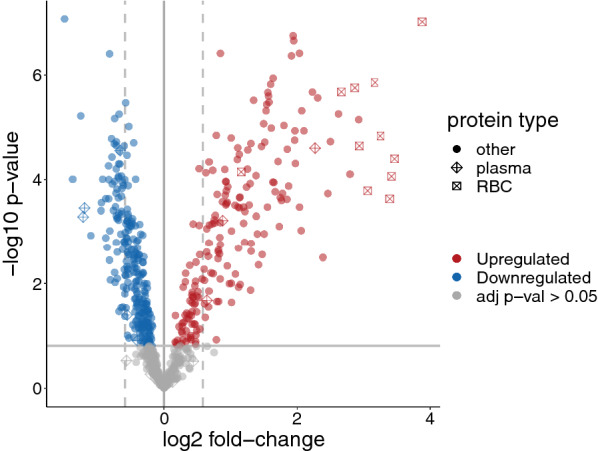
Volcano plot of PDR vs. control. Differential expression analysis compared PDR samples to control samples and included the subset of proteins measured in all samples (see “Methods” for details). The dashed vertical lines highlight linear fold-changes greater than 1.5; the solid horizontal line highlights the Storey adjusted p-value cutoff of 0.05. Erythroid (RBC), plasma, and other proteins are differentiated by shape

Differential expression analysis compared expression in 22 PDR samples to 10 control samples across the subset of 727 proteins measured consistently in all samples. Pooled technical replicates were excluded from differential analysis. A subset of 451 (62%) proteins showed statistical significance (Storey adjusted moderated p-value < 0.05); of those, 242 (33%) had linear fold changes above 1.5. The volcano plot shows that the 15 select plasma proteins noted in heatmaps above were evenly distributed among the other proteins, but the 12 erythroid (RBC) proteins were all highly upregulated in PDR samples (Fig. 11).

### Pathway analyses and biological significance of differential expression analysis

To infer biological meaning from the differentially expressed proteins in control and PDR samples, pathway analysis was performed using multiple methods. iPathway Guide yielded p-values scoring overrepresentation and perturbation. The overrepresentation score is based on pathway component enrichment, while the perturbation score measures expression changes across pathway topology to determine whether the pathway is abnormally disturbed [45]. Ingenuity Pathway Analysis (IPA) also scores pathway perturbation, but predicts the direction of perturbation, i.e., whether the pathway is activated or inhibited. The degree of perturbation is represented by a z-score, with positive z-scores signifying pathway activation and negative z-scores indicating pathway inhibition.

Following an FDR correction, iPathway Guide demonstrated statistically significant overrepresentation of “metabolic pathways”, “carbon metabolism”, and “glycolysis/gluconeogenesis” in the overall PDR versus control comparison group. These pathways were also seen in subphenotype comparisons (Table 3).

**Table 3:** iPathway guide results

Comparison Group	Pathway	Overrepresentation	Perturbation	DE Genes	All Genes	FDR-corrected ORA p-value
All PDR vs. All CTL	Metabolic pathways	Yes	No	52	83	1.24E−02
All PDR vs. All CTL	Carbon metabolism	Yes	No	16	19	1.93E−02
All PDR vs. All CTL	Glycolysis/gluconeogenesis	Yes	No	13	15	3.27E−02
PDR-L vs. CTL	Metabolic pathways	Yes	No	32	83	3.16E−02
PDR-L vs. CTL	Proteasome	Yes	No	6	7	3.16E−02
PDR-L vs. CTL	Platelet activation	Yes	Yes	7	9	3.16E−02
PDR-L vs. CTL	Glycolysis/gluconeogenesis	Yes	No	9	15	4.60E−02
PDR-L vs. CTL	HIF-1 signaling pathway	Yes	Yes	7	10	4.60E−02
PDR-M vs. CTL	Metabolic pathways	Yes	No	45	83	1.69E−03
PDR-M vs. CTL	Carbon metabolism	Yes	No	15	19	2.97E−03
PDR-M vs. CTL	Glycolysis/gluconeogenesis	Yes	No	12	15	1.11E−02
PDR-M vs. CTL	Pentose phosphate pathway	Yes	No	7	7	1.32E−02
PDR-M vs. CTL	Proteasome	Yes	No	7	7	1.32E−02
PDR-H vs. CTL	Metabolic pathways	Yes	No	57	83	3.48E−03
PDR-H vs. CTL	Carbon metabolism	Yes	No	17	19	1.19E−02

Pathway analysis results obtained using iPathway Guide (Advaita). All individually treated PDR samples were compared to all individually treated experiment 2 control samples (All PDR v.s All CTL). PDR subphenotypes were also compared to all control samples. *CTL, control. DE, differentially expressed. ORA, overrepresentation analysis*

Using a z-score cutoff of magnitude 2, IPA showed activation of “glycolysis I”, “gluconeogenesis I”, “protein kinase A signaling”, “NRF2-mediated oxidative stress response”, and “SPINK1 pancreatic cancer pathway” in the overall PDR versus control comparison. By contrast, “semaphorin neuronal repulsive signaling pathway”, “IL-15 production”, “LXR/RXR activation”, and “synaptogenesis signaling pathway” were inhibited in this comparison.

### Extracellular vesicle size distribution and abundance

**Fig. 12:**
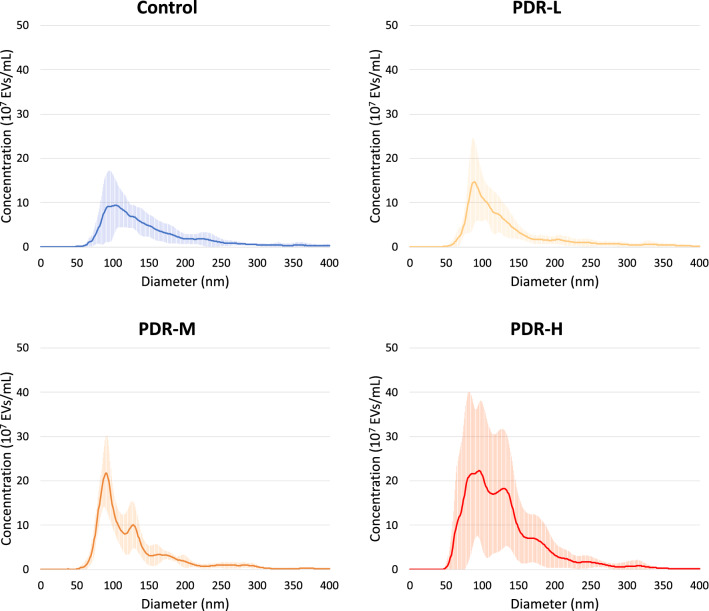
NTA was performed to compare average vesicle concentrations and sizes for each phenotype. Bright lines represent average EV concentration, while error bars are shown in lighter lines above and below this line. Total vesicle abundance was greater in PDR vitreous than that of controls and increased in parallel with increasing ranges of hemoglobin concentration. Across all subphenotype groups, an EV population at an approximate diameter of 90 nm predominates. A second EV population at ~130 nm increases in abundance with increasing hemoglobin concentration

NTA was used to quantify the size distribution and abundances of EVs in vitreous. Prior studies have validated the assumption that nanoparticles measured by NTA do indeed represent EVs [14]. In this context, the term EV refers to any extracellular vesicle, regardless of size or surface markers. NTA of unfractionated vitreous showed differing distributions of EV size and abundance across subphenotypes. Averaged vesicle concentrations and sizes for each subphenotype are shown in Fig. 12. Total vesicle abundance was greater in PDR vitreous than that of controls and increased in parallel with increasing ranges of hemoglobin concentration. Across all subphenotype groups, an EV population at an approximate diameter of 90 nm predominates. A second EV population at ~130 nm is seen to increase in abundance with increasing hemoglobin concentration.

## Discussion

MS-based proteomics is a popular method for interrogating the composition of vitreous in retinal disease states, including PDR. Prior shotgun proteomic studies of PDR vitreous vary greatly in the sample sizes used, which range from one to 74 samples per group [16, 46]; MS methods chosen; and number of proteins identified, ranging from as few as 11 to over 2400 [16, 47]. This study aimed to develop and validate a feasible, rigorous, and scalable method for vitreous proteomic studies through assessment of variability and determination of power. Pathway analyses to infer biological meaning revealed previously unknown alterations that may be implicated in PDR pathogenesis.

Technical variability was nearly absent when performing TMT-MS using a single 10-plex and remained minimal when using multiple plexes. Biological variability was greater than technical variability, as expected, but remained quite low. Normalizing across plexes did not reveal any evident plex bias, underlining the feasibility of applying the described normalization methods to studies examining samples distributed across multiple plexes. Given the number of samples per group required to achieve acceptable power, TMT multiplexing will be critical to scale up experiments while minimizing batch effects. Bridging across plexes using pool samples may prove to be an integral technique to realize sufficient power for differential expression analysis in inherently noisy proteomic data with lower biological effect sizes. Samples within a disease phenotype or subphenotype were similar to one another. One concern that has arisen in vitreous proteomic studies utilizing PDR samples is that blood contamination may skew results [48–51]. A prior study addressed this concern by excluding samples with hemoglobin concentrations > 5 mg/mL (equal to 0.5 g/dL) [50]. Given this concern, we grouped PDR subphenotypes according to hemoglobin concentration in non-depleted vitreous. Samples that were visibly tinted yellow or red had hemoglobin concentrations no higher than 0.0084 g/dL (1786 times lower than the average hemoglobin concentration in blood of 15 g/dL). Therefore, our hemoglobin cutoff is more conservative than what has been reported in prior literature. To further address the concern of blood contamination, we visualized the distribution and hierarchical clustering of abundant plasma proteins and proteins highly expressed in erythrocytes relative to other cell types. No discernable pattern was seen in the distribution of the proteins across samples, phenotypes, or subphenotypes, and these proteins did not appear to drive sample clustering in any way. Thus, it is unlikely that blood contamination contributed in any significant way to the vitreous proteome in PDR.

Due to the high degree of similarity in terms of pathways differentially expressed across the three PDR subphenotypes compared to controls and the above findings on power, the following discussion focuses on the overall comparison group of all PDR samples versus all controls. Only those pathways with statistically significant differential expression and/or activation status were included in the analysis, defined as p-value < 0.05 and z-score ≥ magnitude 2. Differential expression is reported according to the overrepresentation p-value generated by iPathway guide, while activation status is given in terms of z-score generated by IPA.

Pathways centering on metabolism were both overrepresented and activated in PDR vitreous relative to controls. “Metabolic pathways”, “carbon metabolism”, and “glycolysis/gluconeogenesis” were differentially expressed in PDR vitreous relative to controls, with the vast majority of pathway components being upregulated (Table 3). In addition to being overrepresented, glycolytic and gluconeogenic pathways were predicted to be activated (Table 4).

**Table 4:** Ingenuity pathway analysis results

Comparison group	Pathway	p-value	Z-score
All PDR vs. All CTL	Glycolysis I	2.51E−08	3.32
All PDR vs. All CTL	Gluconeogenesis I	8.91E−07	3.16
All PDR vs. All CTL	Protein kinase A signaling	3.63E−02	2.98
All PDR vs. All CTL	NRF2-mediated oxidative stress response	1.05E−02	2.24
All PDR vs. All CTL	SPINK1 pancreatic cancer pathway	3.63E−04	2.12
All PDR vs. All CTL	Semaphorin neuronal repulsive signaling pathway	5.75E−03	− 2.53
All PDR vs. All CTL	IL-15 production	9.12E−04	− 2.71
All PDR vs. All CTL	LXR/RXR activation	1.58E−37	− 2.95
All PDR vs. All CTL	Synaptogenesis signaling pathway	2.82E−08	− 3.02
PDR-L vs. CTL	Protein kinase A signaling	3.63E−02	3.44
PDR-L vs. CTL	Glycolysis I	2.51E−08	3.32
PDR-L vs. CTL	Gluconeogenesis I	8.91E−07	3.16
PDR-L vs. CTL	GP6 signaling pathway	1.00E−10	2.40
PDR-L vs. CTL	NRF2-mediated oxidative stress response	1.05E−02	2.24
PDR-L vs. CTL	Dendritic cell maturation	1.15E−03	2.14
PDR-L vs. CTL	BAG2 signaling pathway	4.27E−02	2.00
PDR-L vs. CTL	Extrinsic prothrombin activation pathway	1.58E−11	− 2.00
PDR-L vs. CTL	Dermatan sulfate degradation (Metazoa)	4.27E−03	− 2.00
PDR-L vs. CTL	IL-15 production	9.12E−04	− 2.11
PDR-L vs. CTL	Chondroitin sulfate degradation (Metazoa)	4.79E−04	− 2.24
PDR-L vs. CTL	Synaptogenesis signaling pathway	2.82E−08	− 2.65
PDR-L vs. CTL	LXR/RXR activation	1.58E−37	− 3.24
PDR-M vs. CTL	Glycolysis I	1.51E−10	3.32
PDR-M vs. CTL	Gluconeogenesis I	3.24E−09	3.16
PDR-M vs. CTL	SPINK1 pancreatic cancer pathway	5.89E−04	2.83
PDR-M vs. CTL	NRF2-mediated oxidative stress response	1.78E−02	2.24
PDR-M vs. CTL	LXR/RXR activation	1.58E−37	− 2.06
PDR-M vs. CTL	Glioma invasiveness signaling	3.02E−02	− 2.45
PDR-M vs. CTL	Synaptogenesis signaling pathway	7.76E−08	− 2.65
PDR-M vs. CTL	IL-15 production	1.62E−03	− 2.71
PDR-M vs. CTL	Semaphorin neuronal repulsive signaling pathway	8.51E−03	− 3.16
PDR-H vs. CTL	Glycolysis I	2.51E−08	3.32
PDR-H vs. CTL	Gluconeogenesis I	8.91E−07	3.16
PDR-H vs. CTL	Protein kinase A signaling	3.63E−02	2.98
PDR-H vs. CTL	NRF2-mediated oxidative stress response	1.05E−02	2.24
PDR-H vs. CTL	SPINK1 pancreatic cancer pathway	3.63E−04	2.12
PDR-H vs. CTL	Leukocyte extravasation signaling	3.89E−02	2.11
PDR-H vs. CTL	Complement system	2.00E−31	2.07
PDR-H vs. CTL	IL-15 production	9.12E−04	− 2.11
PDR-H vs. CTL	LXR/RXR activation	1.58E−37	− 2.36
PDR-H vs. CTL	Semaphorin neuronal repulsive signaling pathway	5.75E−03	− 2.53
PDR-H vs. CTL	Synaptogenesis signaling pathway	2.82E−08	− 2.65

Pathway analysis results obtained using Ingenuity Pathway Analysis (IPA; Qiagen). All individually treated PDR samples were compared to all individually treated experiment 2 control samples (All PDR v.s All CTL). PDR subphenotypes were also compared to all control samples

*CTL* control, *DE* differentially expresse*d*

Carbohydrate metabolism, including glycolytic and gluconeogenic pathways, is dysregulated in diabetes. Glycolysis converts glucose into lactate and releases ATP and reducing equivalents, whereas gluconeogenesis is a reversal of this pathway that generates glucose from non-carbohydrate precursors. These pathways are encompassed within “carbon metabolism”, which also includes other carbon utilization pathways such as the pentose phosphate pathway and citric acid cycle. “Metabolic pathways” encompasses all of the differentially expressed genes in “glycolysis/gluconeogenesis” and “carbon metabolism.”

The duration and degree of hyperglycemia in persons with diabetes are associated with the development and progression of DR [52], and intensive management of blood glucose reduces the risk of DR development and progression [53–61]. Recent evidence indicates that abnormal flux through the glycolysis pathway leads to the activation of several pathways known to be involved in the pathogenesis of complications of diabetes. Direct assessment of retinal metabolism using radiolabeled glucose revealed modest upregulation of glycolysis in the BKS *db/db* mouse model of type 2 diabetes at 24 weeks of age [62]. However, similar studies in a rat model of insulin-deficient diabetes showed no meaningful increase in retinal glycolysis [63]. The overrepresentation of metabolic pathway members in PDR vitreous may similarly indicate disruption of glucose homeostasis due to hyperglycemia.

A prior proteomic study of PDR vitreous by Gao et al. identified elevated “metabolic pathway” components carbonic anhydrase 1 and 2 (CA1, CA2) [49], which reversibly hydrate carbon dioxide as part of pH and fluid balance and were the most upregulated proteins in this pathway in the current study. Gao et al. found levels of CA1 and CA2 to be several times higher in PDR vitreous relative to that of non-diabetic controls. Further analyses of CA1 and CA2 indicated they may increase permeability of the retinal vasculature, with the actions of CA1 being additive to those of vascular endothelial growth factor (VEGF) [49]. Thus, CA1 and CA2 may represent specific metabolic pathway members that contribute to PDR pathogenesis.

“Protein kinase A (PKA) signaling” was also activated in PDR vitreous. This pathway has diverse regulatory activity, modulating growth and development, memory, and metabolic functions. PKA regulates angiogenesis in the developing retina; its inhibition in mice caused vascular defects via an increase in the number of endothelial tip cells, resulting in hypersprouting [64]. Similarly, a later study demonstrated that PKA reduced endothelial sprouting capacity [65]. These angiogenic regulatory effects may reflect an attempt by the retina to modulate revascularization in the setting of DR. PKA-dependent pathways also augment retinal ganglion cell regeneration [66].

“Nuclear factor-erythroid 2-related factor 2 (Nrf2)-mediated oxidative stress response” was activated in PDR vitreous relative to controls and is a widely studied mediator of the cellular response to oxidative stress. Nrf2 is a transcription factor that, when activated, leads to transcription of antioxidant enzymes and other proteins involved in detoxification. Because of the high rate of oxygen consumption in the retina relative to other tissues, it is especially vulnerable to oxidative stress [67, 68]. Given this vulnerability, it is not surprising that oxidative stress is involved in various mechanisms underlying DR pathogenesis [69, 70]. Reactive oxygen species (ROS) are the main source of oxidative stress and are produced physiologically during carbohydrate metabolism. When the production of ROS cannot be balanced with antioxidant mechanisms, ROS accumulate and induce DNA damage and inflammation, stimulating VEGF production [70]. Nrf2 signaling serves as an attempt to offset these pathological changes. Xu et al. demonstrated that knockout of *Nrf2* in a diabetic mouse model resulted in early blood-retina barrier dysfunction and declining neural function [71]. A study of diabetic rats and human donor retinas demonstrated increased Nrf2 levels, but decreased Nrf2 activity due to increased binding with its inhibitor, Keap1, preventing its translocation to the nucleus for transcription of antioxidant response elements [72]. The increased activation of Nrf2 signaling in PDR vitreous may indicate an effort, though possibly unsuccessful, to restore the balance of antioxidant molecules in the context of increased ROS.

“Serine protease inhibitor, Kasal type 1 (SPINK1) pancreatic cancer pathway” was activated in PDR vitreous. SPINK1 is known for its roles in inhibiting pancreatic trypsin in cases of premature trypsinogen activation and in familial forms of pancreatitis. More recently, however, SPINK1 has been recognized as a possible acute phase reactant [73] and growth factor and has specifically been shown to stimulate endothelial cell growth [74]. Though the role of SPINK1 signaling in the retina is yet unknown, a recent study demonstrated a higher incidence of DR in individuals with fibrocalculous pancreatic diabetes, a form of diabetes mellitus often associated with SPINK1 mutations, relative to individuals with type 2 diabetes mellitus [75]. Further research is needed to elucidate the role of SPINK1 in DR.

In contrast to activated pathways, “semaphorin neuronal repulsive signaling pathway” was inactivated in the PDR group relative to controls. Specifically, the semaphorins sema3A, sema3F, and sema6A were present at decreased levels. Semaphorins have dual roles in regulating both repulsive neuronal guidance during development and angiogenesis. Sema3A and sema3F serve an anti-angiogenic role in retinal and other tissues. Sema3A is produced by retinal ganglion cells under hypoxic conditions and decreases endothelial cell migration, directing neovascularization away from ischemic retina and toward the vitreous. However, intravitreally delivered recombinant sema3A prevents neovascularization into the vitreous [76]. Thus, the location of sema3A determines the direction of its anti-angiogenic effects. The decreased levels of sema3A in PDR vitreous in the current study are in line with these findings, as PDR is characterized by neovascularization into the vitreous. Sema3F has also been shown to have anti-angiogenic functions, mainly in the outer retina, where it is almost singularly expressed. Reduced sema3F levels have been identified in retinal pigment epithelium derived from human donors with a history of neovascularization of the outer retina [77]. Additional studies are needed to determine whether sema3F plays a role in neovascularization of the inner retina, as occurs in PDR. Recently, sema3F was shown to suppress VEGF-induced endothelial cell proliferation with a higher efficacy than anti-VEGF antibody treatment; this effect was observed at a sema3F concentration tenfold lower than that of VEGF [78]. Sema6A also decreases endothelial cell migration in a dose-dependent manner [79]. Decreased semaphorin signaling in PDR vitreous may be one factor permitting the extension of retinal neovascularization into the vitreous.

“Interleukin (IL)-15 signaling” activation was decreased in PDR vitreous relative to controls. IL-15 regulates natural killer cells and T lymphocytes and is produced by diverse cell types, including macrophages, fibroblasts, and nerve cells. More recently, roles for IL-15 in metabolism have been elucidated, specifically in the context of obesity. Obesity is a key player in the development of insulin resistance, a phenomenon that characterizes the pathogenesis of type 2 diabetes mellitus. Sun and Liu found that transfer of the IL-15 gene in high-fat diet-induced obese mice prevented weight gain, lessened the development of hepatic steatosis, and improved glucose homeostasis [80]. Transfer of the IL-15 gene along with its soluble receptor had the same effects, along with improving insulin sensitivity [81]. Similarly, Barra et al. [82] found that delivery of the IL-15 gene to high-fat diet-induced obese mice increased sensitivity to insulin and better responses to a glucose challenge relative to both untreated high-fat diet mice and low-fat diet lean controls. IL-15 may also have insulin-independent effects on glucose metabolism. A recent study demonstrated that IL-15 improved glucose metabolism by activating AMP-activate protein kinase (AMPK) and increasing glucose transporter type 4 (GLUT4) translocation to the skeletal muscle membrane [83]. AMPK-mediated GLUT4 translocation is induced by exercise in a mechanism independent of insulin [84, 85] and thus may mitigate the effects of insulin resistance. Further research is needed in order to determine whether these properties are generalizable to retinal tissue, but it is possible that the decrease in IL-15 signaling reflects the impaired glucose and insulin responses that ultimately precipitate diabetes complications such as retinopathy.

“Liver X receptor (LXR)/retinoid X receptor (RXR) activation” was also inhibited in PDR vitreous. RXRs and LXRs are nuclear receptors that form heterodimers to exert transcriptional regulation. RXRs facilitate the actions of retinoids, while LXR acts to increase cholesterol efflux. The LXR/RXR heterodimer has regulatory functions on both metabolic and inflammatory processes. Studies evaluating the actions of the LXR/RXR heterodimer in diabetes are lacking, but ample research exists on each individual receptor. Multiple studies have demonstrated a glucose-lowering effect of RXR agonists. RXR agonists have been shown to lower serum glucose levels [86, 87] and increase insulin sensitivity [87] in diabetic animal models. RXR also increases both insulin-dependent and independent glucose uptake in skeletal muscle [88]. Given these glucose-lowering effects of RXR agonists, the decreased activity of RXR in PDR vitreous may contribute to dysregulated retinal glucose metabolism. LXR, a cholesterol-modulating receptor that also functions in inflammation, may influence the development of diabetes and its complications through actions on multiple cell types. In a mouse model of diabetes, an LXR agonist improved the function of endothelial cell precursors responsible for vascular repair and reduced expression of a marker of neurodegeneration [89], suggesting involvement in both vascular and neural effects of diabetes. LXR activation has also been shown to block hyperglycemia-induced endothelial cell senescence, potentially protecting against the atherosclerotic processes that are accelerated in diabetes [90]. Treatment of diabetic animals with an LXR agonist resulted in neuroprotective effects [91], further suggesting a role in the neurodegenerative aspect of diabetes. In line with decreased activity of LXR/RXR activation in PDR vitreous in the current study, decreased LXR was observed in retinal tissue from both diabetic mice and diabetic human donors [92]. The decreased LXR/RXR activation in PDR the current study is thus in line with the microvascular and neurodegenerative processes that characterize diabetic retinopathy.

“Synaptogenesis signaling pathway” was also inhibited in PDR vitreous. Synaptogenesis refers to the formation of neural synapses and is mediated by interactions between diverse adhesion molecules. In ocular development, synaptogenesis plays a role in synchronizing the timing of retinal synapse formation with eye opening [93]. Studies specifically examining synaptogenesis in the adult retina in diabetes are lacking, but synaptogenesis is known to be a continuous and dynamic modulator of neural circuitry in the adult brain [94]. Dysfunctional synaptogenesis is both a cause and an outcome of various neurodegenerative and neurodevelopmental central nervous system disorders [95]. Neurodegenerative changes in the retina precede the microvascular injury of clinically detectable diabetic retinopathy [96–99]. Consistent with prior literature, it is possible that decreased synaptogenesis signaling in PDR vitreous contributes to or results from the neurodegenerative changes that occur in early DR pathogenesis.

Collectively, these data reveal profound alterations in ocular metabolism, inflammatory processes, and neurotrophic pathways in patients with PDR. These findings are consistent with the late stage of DR in which most of the patients had previously undergone panretinal laser photocoagulation and/or intravitreal anti-vascular endothelial cell growth factor treatments. These patients exhibit both vascular and neural retinal degeneration and have developed neovascular and fibrotic responses that lead to the need for therapeutic vitrectomy. Though metabolic processes are not generally viewed as primary drivers of PDR pathogenesis, it is possible that their overrepresentation in PDR represents a previously overlooked component of disease pathogenesis. That metabolic processes predominated over vascular proliferation pathways may reflect prior treatment of all PDR patients with anti-VEGF and/or panretinal photocoagulation, both of which aim to halt neovascularization.

We carefully examined the potential role of blood on the proteome profile by phenotyping the samples by color and hemoglobin concentration. Remarkably, plasma and erythrocyte-derived proteins did not distinguish PDR proteomic profiles from the non-diabetic controls. The process of depleting the most abundant plasma-derived proteins prior to MS analysis was a standard feature of our protocol and is important to enable detection of low abundance retina-derived proteins that are likely involved in the pathogenesis of PDR.

### Potential role of extracellular vesicles

In addition to the components of the vitreous proteome discussed above, we have previously shown that human vitreous contains an abundant population of EVs [14]. In the current study, EVs were more abundant in PDR vitreous than that of control, and distinct size populations varied with subphenotype. The increased population of larger diameter EVs seen across subphenotypes with increasing hemoglobin concentration may represent a uniquely functioning subset of EVs related to PDR pathogenesis or severity. EVs are secreted by all cell types and function in cell–cell communication, playing critical roles in both physiological and pathological processes. These vesicles contain diverse biomolecules indicative of the processes occurring in their parent cells at the time of secretion and are therefore rich with information about the tissues from which they derive [100, 101]. Although the specific cells of origin of vitreous EVs remain unknown, it is plausible that they derive from multiple cell types in the adjacent tissues, including cells of the retina. Therefore, vitreous EV analysis may yield critical information about retinal disease states. Many of the above pathways have been shown to be mediated, in part, by EVs. For example, EVs play an emerging role in metabolism and metabolic disease. The majority of enzymes of the glycolysis pathway are among the top 100 most commonly identified proteins in exosomes [102], a subset of EVs. In certain systems, EVs independently generate ATP via glycolysis [103]. EVs also impede insulin signaling and precipitate insulin resistance in adipose tissue [104, 105]. Due to their high cholesterol content, EVs may serve as an additional strategy to reduce the cellular lipid burden in cholesterol-overloaded conditions, helping to preserve cholesterol homeostasis [106]. Recently, EVs secreted by liver cells were shown to regulate adipose and lipid production in recipient adipocytes [107]. In addition to metabolic functions, EVs play roles in angiogenesis via multiple pathways. EVs are able to both deliver angiogenic molecules, including VEGF, from cell to cell [108] and to induce expression of VEGF transcripts [109]. An EV-associated form of VEGF was shown to possess increased potency in terms of VEGF receptor activation in recipient cells [110]. Other angiogenic signaling molecules, such as the angiopoietins and Wnt pathway members, are also present in EVs [111–118]. EVs also possess protective properties against oxidative stress. EVs have been shown to alleviate oxidative stress in animal models via Nrf2 activation [119] and by lessening myeloperoxidase and ROS activities [120]. In addition to modulating oxidative stress, EVs also have the capacity to enhance or decrease inflammatory processes. EVs released by cells exposed to inflammation exert anti-inflammatory effects via the cyclooxygenase/prostaglandin E2 pathway [121]. Contrarily, cells exposed to lipids released EVs that led to pro-inflammatory changes in recipient cells [122]. Emerging evidence also points to a role for EVs in synaptic plasticity. Wnt pathway members are involved in synapse formation and plasticity, and Wnt members and their binding proteins are transferred across neuromuscular synapses in a mechanism requiring EVs [116]. Other proteins with known roles in synaptic plasticity also require EVs for their trans-synaptic transport and function [123, 124]. Thus, EVs are abundant in vitreous and play known roles in the processes reflected in the above pathway analysis, namely metabolism, angiogenesis, oxidative stress, inflammation, and synaptogenesis. Further investigation is needed to determine the functional role of EVs in the vitreous and the degree to which they may be involved in the pathways discussed here.

The current study underlines the importance of taking statistical power into account when designing vitreous proteomics studies. The use of mass spectrometry-based analysis enabled unbiased identification of signaling pathways, an advantage over prior studies of vitreous utilizing only targeted approaches such as enzyme-linked immunosorbent assays or western blots. This approach facilitated identification of both upregulated and downregulated pathways, the latter of which is often brushed over in the literature. The current study also has the advantage of including an activation z-score to show directional pathway involvement, whereas prior studies have focused mainly on pathway enrichment only. Further, the results presented here are the first to validate the use of bridging samples to scale up sample size to achieve sufficient power. These results also show that arranging such samples in plexes of 10 accomplishes comparable proteomic depth of coverage at 1/10^th^ the cost of non-plexed experiments, increasing the feasibility of larger scale vitreous proteomics studies.

A limitation of this study is its examination of only a single retinal disease. Although the findings from these data can be applied to other diseases, precise determination of the ideal sample size for analysis of particular disease phenotypes will need to be assessed on an individualized basis. Nonetheless, the current data can serve as a useful guide for both interpreting the statistical rigor of prior vitreous proteomic studies and for estimating the necessary sample size in future studies.

Future studies are needed to determine whether sufficient power can be achieved at similar sample sizes in vitreous proteomic studies examining different retinal diseases.

## Conclusions

The current study demonstrated minimal technical variability and low biological variability using TMT-MS to interrogate 10-plexes of human vitreous samples. Normalization proved a feasible method for examining samples across multiple plexes, underlining the scalability of this technique. Inclusion of bridging samples in each plex may increase the power that can be achieved in multiplexed MS experiments utilizing biological samples. Pathway analysis revealed pathways involved in processes that are known to be dysregulated in diabetes and/or DR, including carbohydrate and lipid metabolism, angiogenesis, oxidative stress, inflammation, and neural processes. Some of the pathways within these categories have not been previously studied in the context of DR; therefore, the proteomic pipeline described here may facilitate discovery of novel players in PDR pathogenesis. Further, EVs are known to be involved in the above pathways in other systems in both physiological and pathological states, and PDR vitreous contains EVs in greater amounts than does control vitreous. Interrogation of the EV-associated vitreous proteome may therefore prove to be a valuable method for uncovering dysregulated pathways in PDR.

## Supplementary Information

**Additional file 1**. Supplementary material detailing inputs, protein sets, and analysis results from experiments 1 and 2 can be found here.

## Data Availability

Mass spectrometry proteomics data have been deposited to the ProteomeXchange Consortium via the PRIDE partner repository with the dataset identifier PXD025986. All other Additional files generated during the current study are available at https://github.com/umich-brcf-bioinf-projects/Oculomics_tomwgard_CU3-power_analysis.

## Abbreviations

AMD: Age-related macular degeneration
AMPK: AMP-activated protein kinase
CA1: Carbonic anhydrase 1
CA2: Carbonic anhydrase 2
DR: Diabetic retinopathy
ERM: Epiretinal membrane
EV: Extracellular vesicle
FDR: False discovery rate
GLUT4: Glucose transporter type 4
IL: Interleukin
IPA: Ingenuity pathway analysis
IQR: Interquartile range
IRB: Institutional review board
LC: Liquid chromatography
LIMMA: Linear model for microarray analysis
LXR: Liver X receptor
MAD: Median absolute deviation
MH: Macular hole
MS: Mass spectrometry
Nrf2: Nuclear factor erythroid 2-related factor 2
NTA: Nanoparticle tracking analysis
PCA: Principal component analysis
PDR: Proliferative diabetic retinopathy
PKA: Protein kinase A
PSM: Peptide to spectrum match
Q: Quantile
RBC: Red blood cell
ROS: Reactive oxygen species
RXR: Retinoid X receptor
SPINK1: Serine peptidase inhibitor, Kazal type 1
TEAB: Triethylammonium bicarbonate
TMT: Tandem mass tag
VEGF: Vascular endothelial growth factor
