# Supplementary material for: A validated analysis pipeline for mass spectrometry-based vitreous proteomics: new insights into proliferative diabetic retinopathy
Source: Clin Proteomics. 2021 Dec 3;18:28. doi: 10.1186/s12014-021-09328-8 (PMC8903510; doi:10.1186/s12014-021-09328-8)
Supplement: Supplementary file 1 — Additional file 1. Supplementary material detailing inputs, protein sets, and analysis results from experiments 1 and 2 can be found here. [file 12014_2021_9328_MOESM1_ESM.zip › Oculomics_tomwgard_CU3-power_analysis-main/outputs/figures/exp1_figures-pairwise_scatter_samples.pdf]

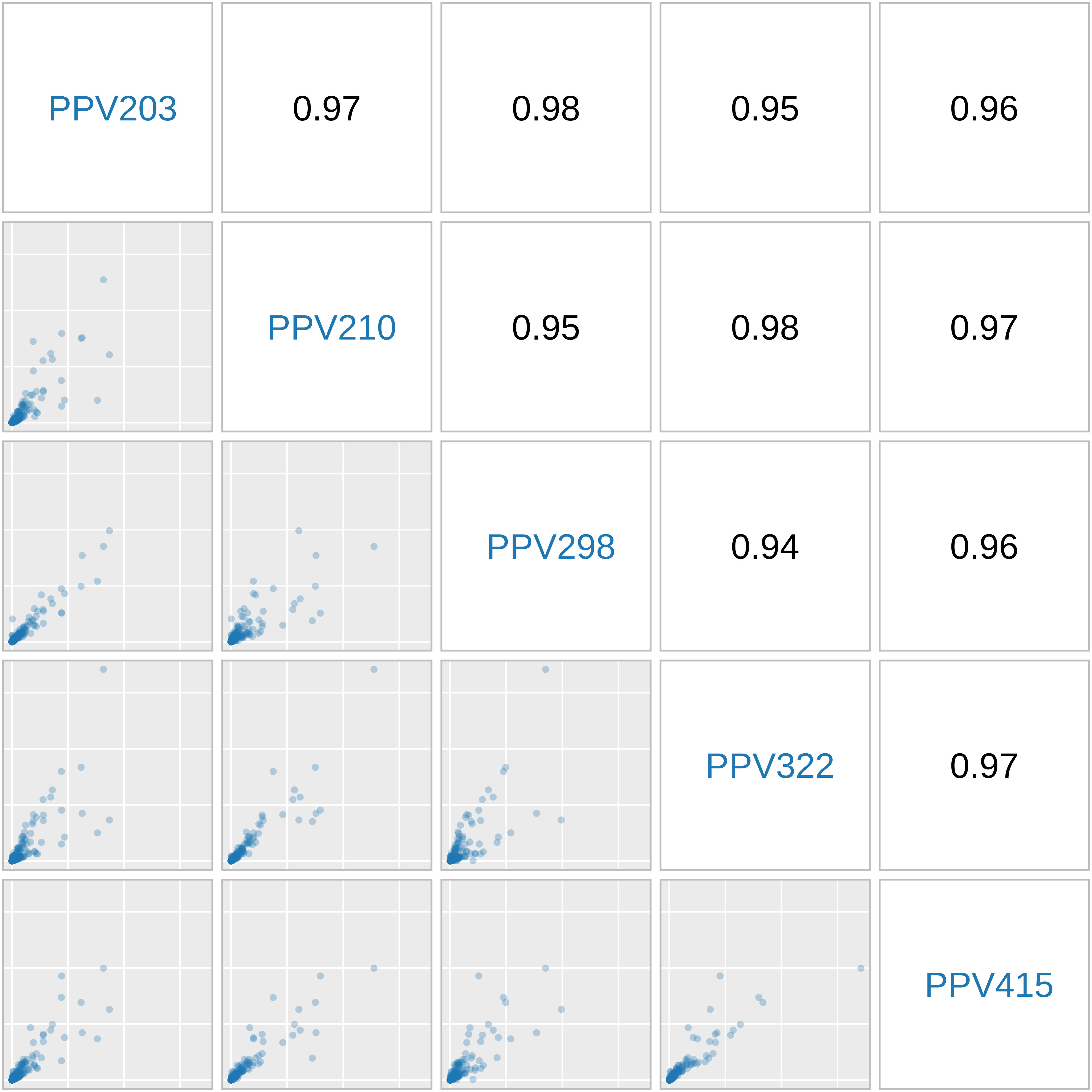
PPV203
0.97
0.98
0.95
0.96
PPV210
0.95
0.98
0.97
PPV298
0.94
0.96
PPV322
0.97
PPV415
