# Supplementary material for: A validated analysis pipeline for mass spectrometry-based vitreous proteomics: new insights into proliferative diabetic retinopathy
Source: Clin Proteomics. 2021 Dec 3;18:28. doi: 10.1186/s12014-021-09328-8 (PMC8903510; doi:10.1186/s12014-021-09328-8)
Supplement: Supplementary file 1 — Additional file 1. Supplementary material detailing inputs, protein sets, and analysis results from experiments 1 and 2 can be found here. [file 12014_2021_9328_MOESM1_ESM.zip › Oculomics_tomwgard_CU3-power_analysis-main/outputs/figures/exp2_figures-normalized_abundance_boxplot.pdf]

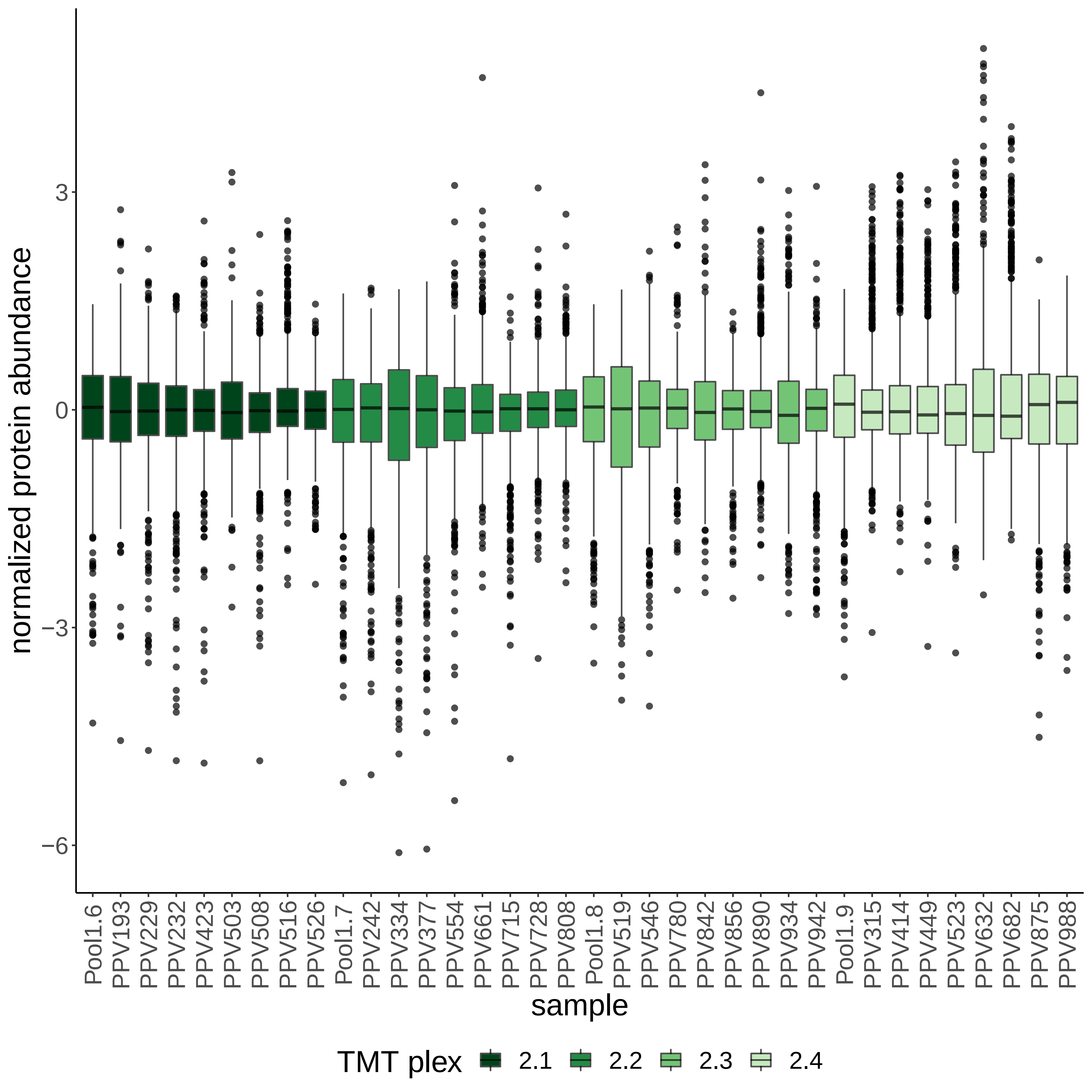
normalized protein abundance
3
0
−3
−6
Pool1.6
PPV193
PPV229
PPV232
PPV423
PPV503
PPV508
PPV516
PPV526
Pool1.7
PPV242
PPV334
PPV377
PPV554
PPV661
PPV715
PPV728
PPV808
Pool1.8
PPV519
PPV546
PPV780
PPV842
PPV856
PPV890
PPV934
PPV942
Pool1.9
PPV315
PPV414
PPV449
PPV523
PPV632
PPV682
PPV875
PPV988
sample
TMT plex
2.1
2.2
2.3
2.4
