# Supplementary material for: A validated analysis pipeline for mass spectrometry-based vitreous proteomics: new insights into proliferative diabetic retinopathy
Source: Clin Proteomics. 2021 Dec 3;18:28. doi: 10.1186/s12014-021-09328-8 (PMC8903510; doi:10.1186/s12014-021-09328-8)
Supplement: Supplementary file 1 — Additional file 1. Supplementary material detailing inputs, protein sets, and analysis results from experiments 1 and 2 can be found here. [file 12014_2021_9328_MOESM1_ESM.zip › Oculomics_tomwgard_CU3-power_analysis-main/outputs/figures/exp2_upset-by_phenotype.pdf]

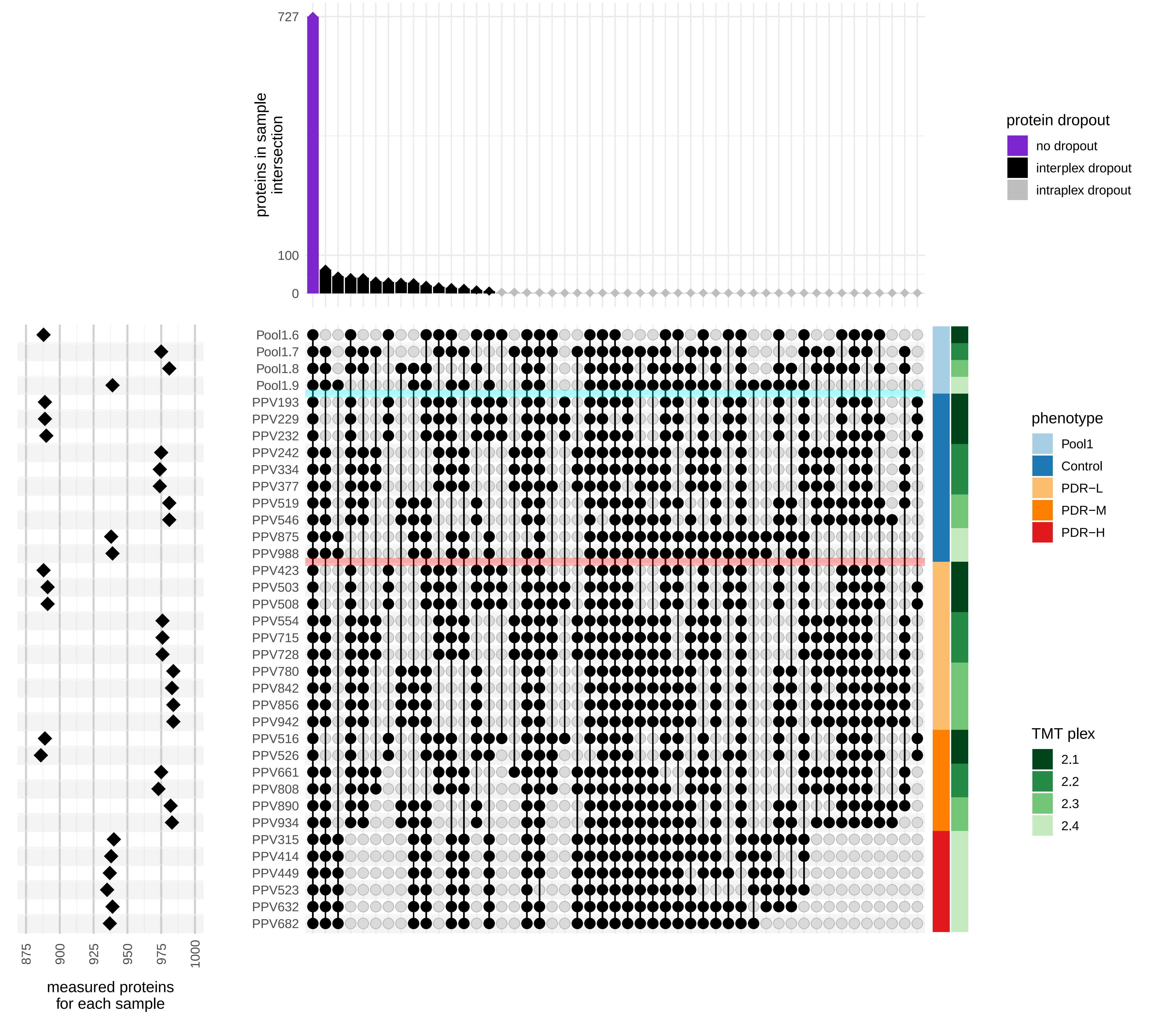

727
100
0
proteins in sample intersection
protein dropout
no dropout
interplex dropout
intraplex dropout
Pool1.6
Pool1.7
Pool1.8
Pool1.9
PPV193
PPV229
PPV232
PPV242
PPV334
PPV377
PPV519
PPV546
PPV875
PPV988
PPV423
PPV503
PPV508
PPV554
PPV715
PPV728
PPV780
PPV842
PPV856
PPV942
PPV516
PPV526
PPV661
PPV808
PPV890
PPV934
PPV315
PPV414
PPV449
PPV523
PPV632
PPV682
phenotype
Pool1
Control
PDR−L
PDR−M
PDR−H
TMT plex
2.1
2.2
2.3
2.4
875
900
925
950
975
1000
measured proteins for each sample
