# Supplementary figures and images for: A validated analysis pipeline for mass spectrometry-based vitreous proteomics: new insights into proliferative diabetic retinopathy
Source: Clin Proteomics. 2021 Dec 3;18:28. doi: 10.1186/s12014-021-09328-8 (PMC8903510; doi:10.1186/s12014-021-09328-8)

## Slide 1
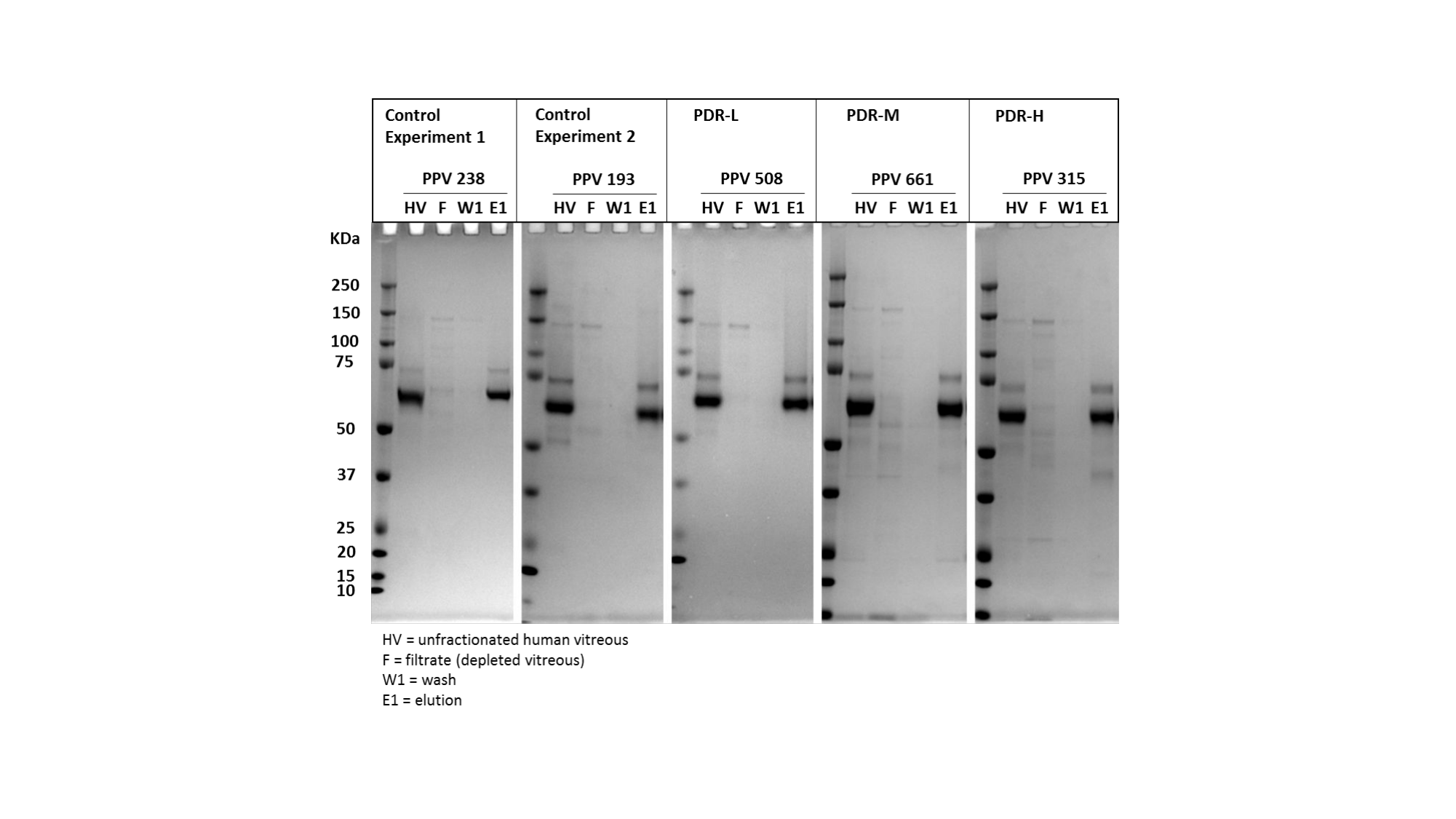

Supplement: Supplementary file 1 — Additional file 1. Supplementary material detailing inputs, protein sets, and analysis results from experiments 1 and 2 can be found here. [file 12014_2021_9328_MOESM1_ESM.zip › Oculomics_tomwgard_CU3-power_analysis-main/inputs/Pre-Post-Depletion_Gels.pptx]

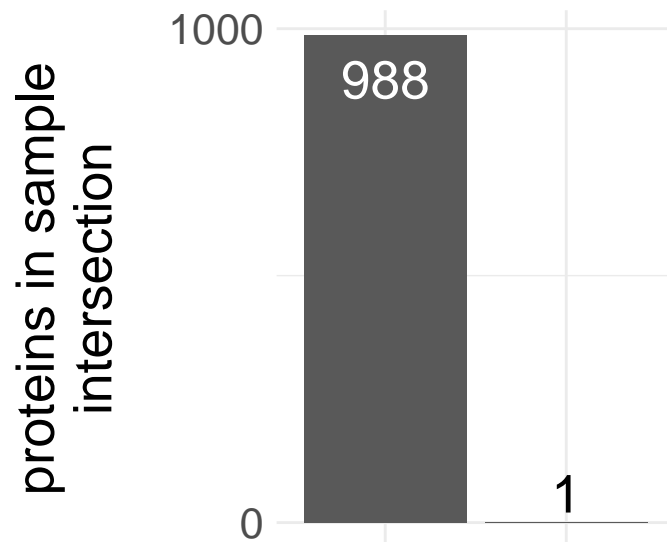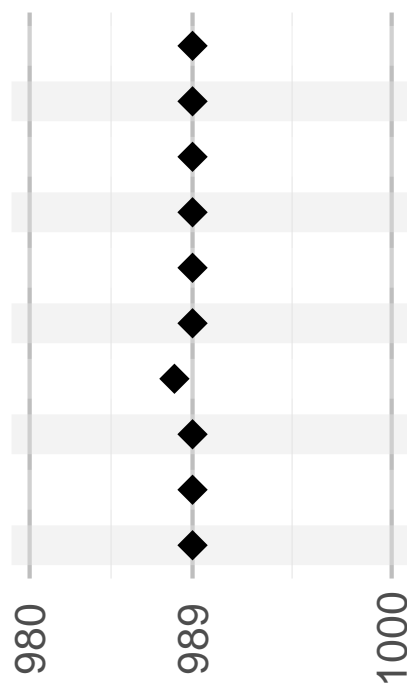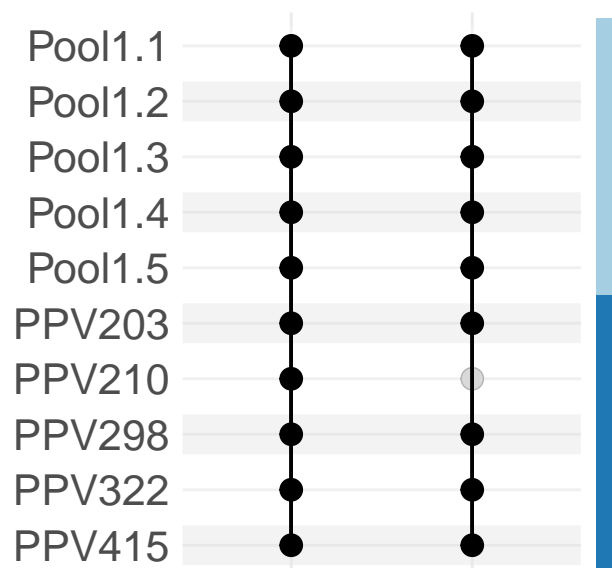

technical replicates  
biological replicates

measured proteins  
for each sample

Supplement: Supplementary file 1 — Additional file 1. Supplementary material detailing inputs, protein sets, and analysis results from experiments 1 and 2 can be found here. [file 12014_2021_9328_MOESM1_ESM.zip › Oculomics_tomwgard_CU3-power_analysis-main/outputs/figures/exp1_upset-by_pool_vs_sample.pdf]

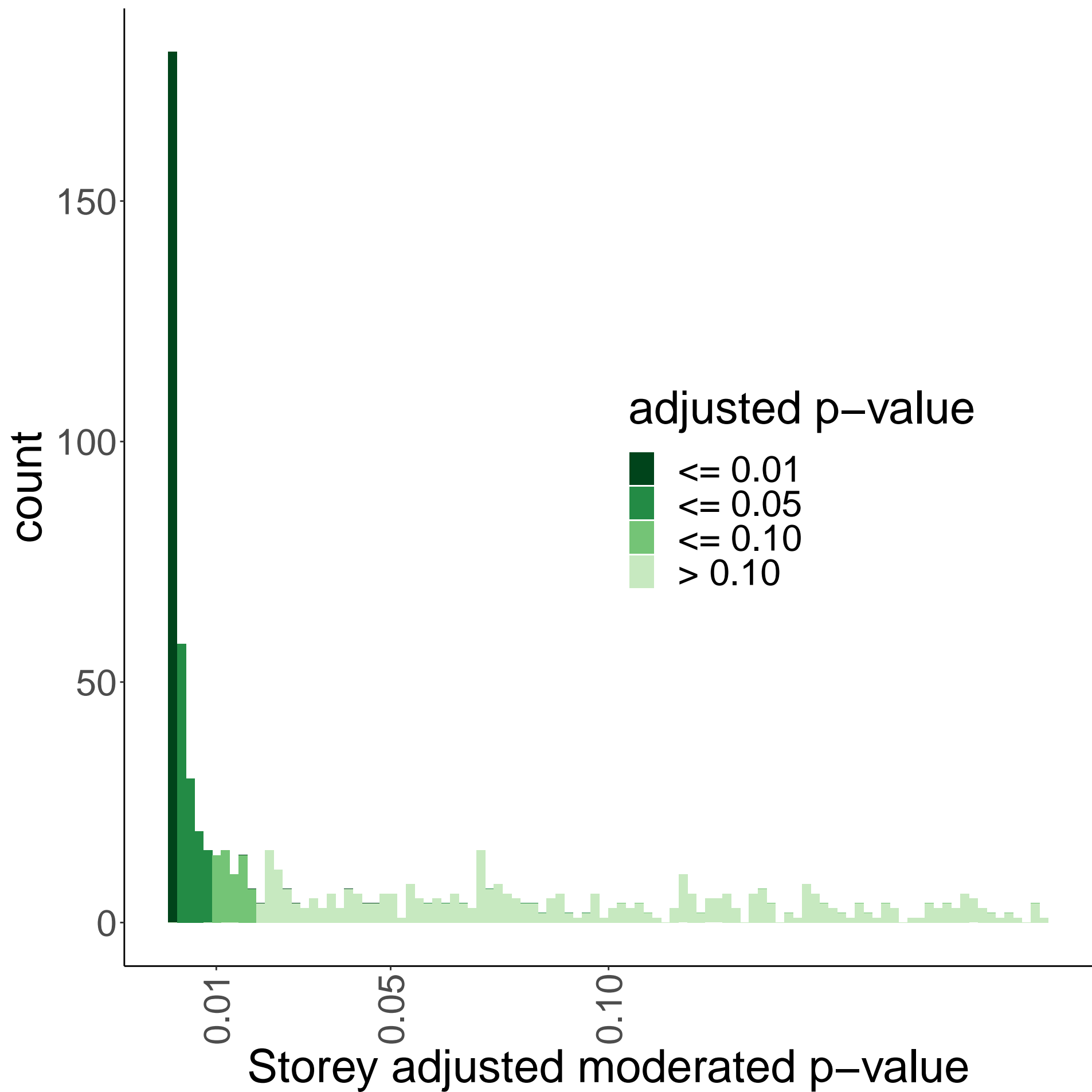

Supplement: Supplementary file 1 — Additional file 1. Supplementary material detailing inputs, protein sets, and analysis results from experiments 1 and 2 can be found here. [file 12014_2021_9328_MOESM1_ESM.zip › Oculomics_tomwgard_CU3-power_analysis-main/outputs/figures/exp2_adjusted_pvalue_histogram.pdf]

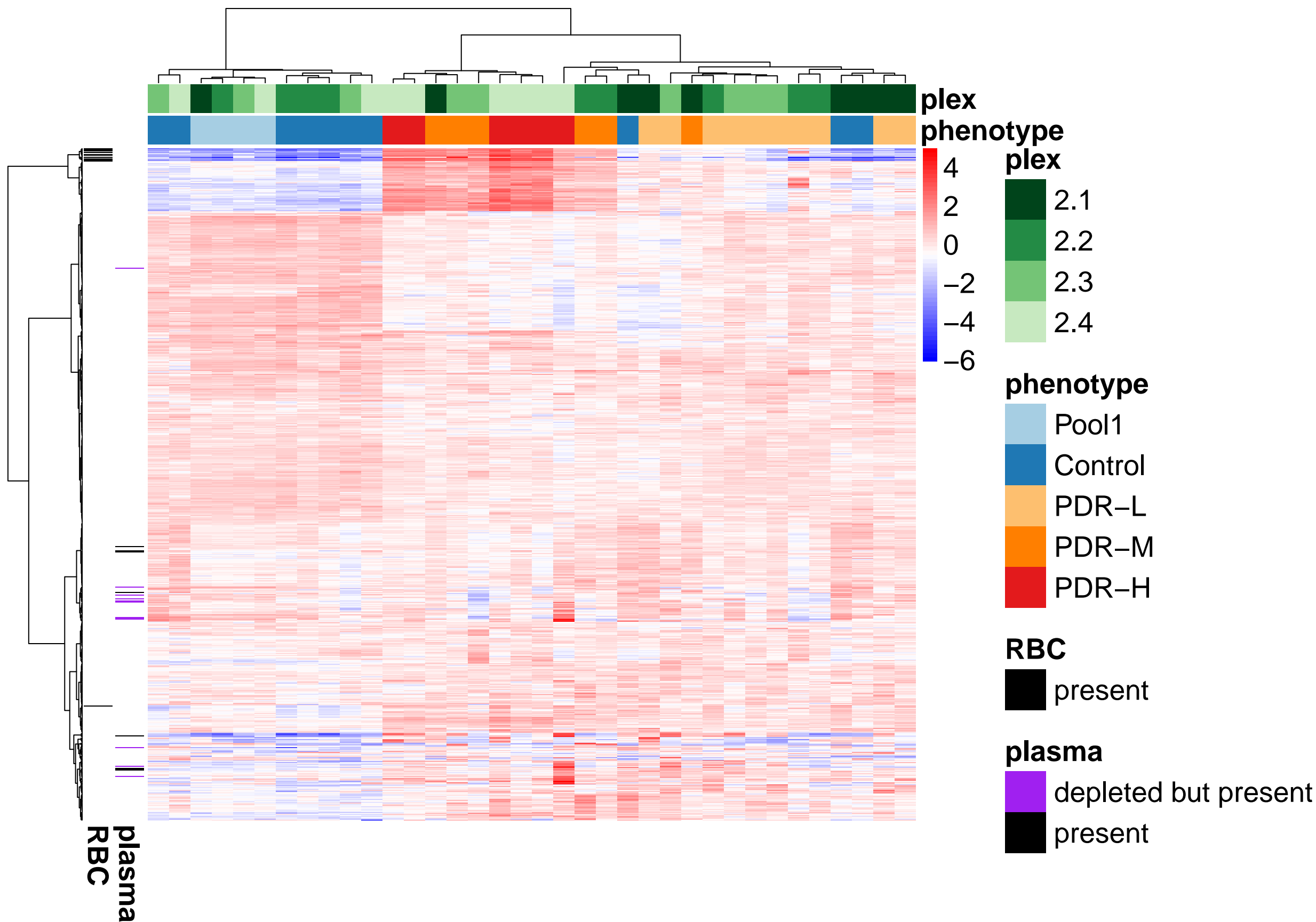

Supplement: Supplementary file 1 — Additional file 1. Supplementary material detailing inputs, protein sets, and analysis results from experiments 1 and 2 can be found here. [file 12014_2021_9328_MOESM1_ESM.zip › Oculomics_tomwgard_CU3-power_analysis-main/outputs/figures/exp2_figures-heatmap_all_annotated.pdf]

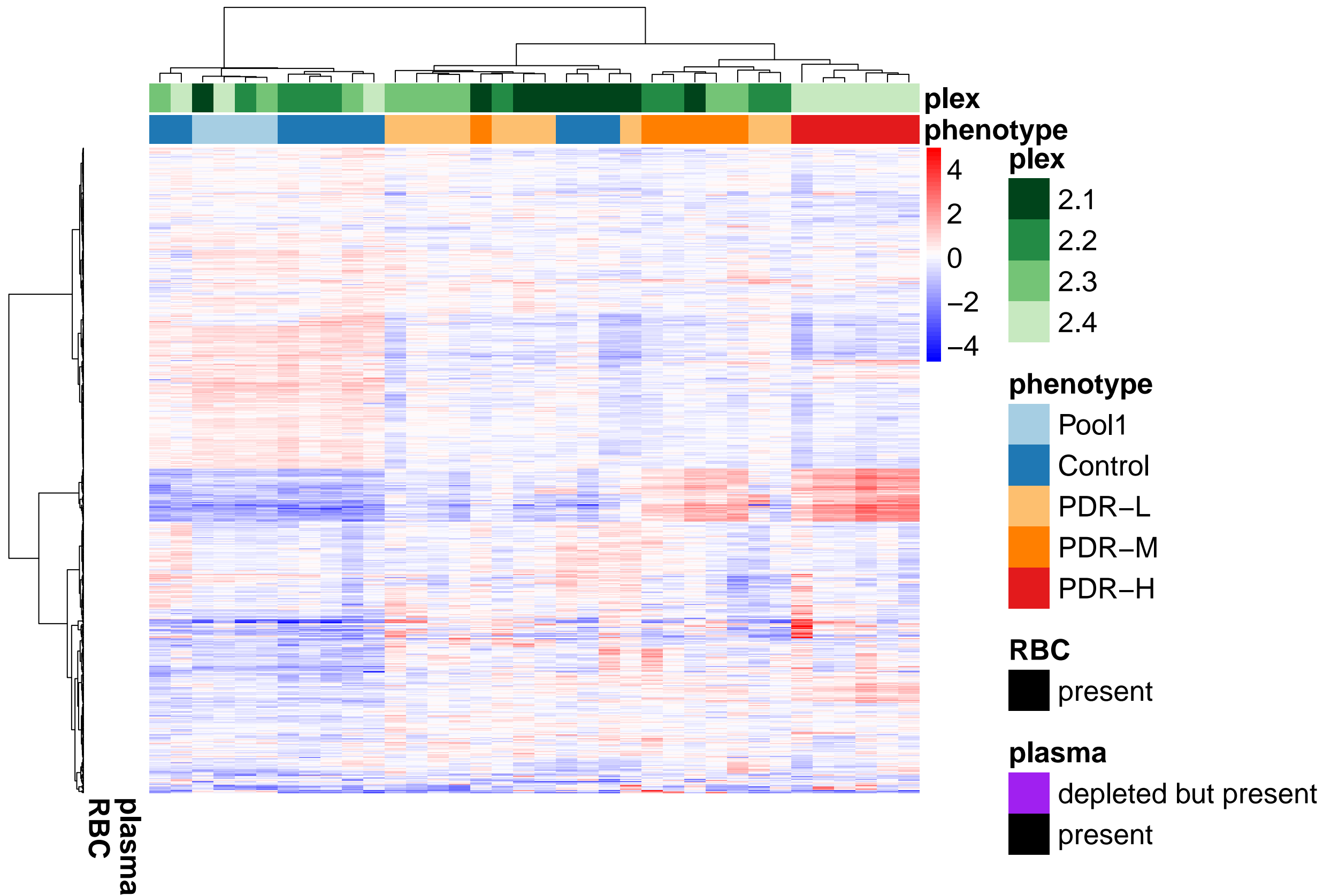

Supplement: Supplementary file 1 — Additional file 1. Supplementary material detailing inputs, protein sets, and analysis results from experiments 1 and 2 can be found here. [file 12014_2021_9328_MOESM1_ESM.zip › Oculomics_tomwgard_CU3-power_analysis-main/outputs/figures/exp2_figures-heatmap_exclude_erythrocyte_plasma_annotated.pdf]

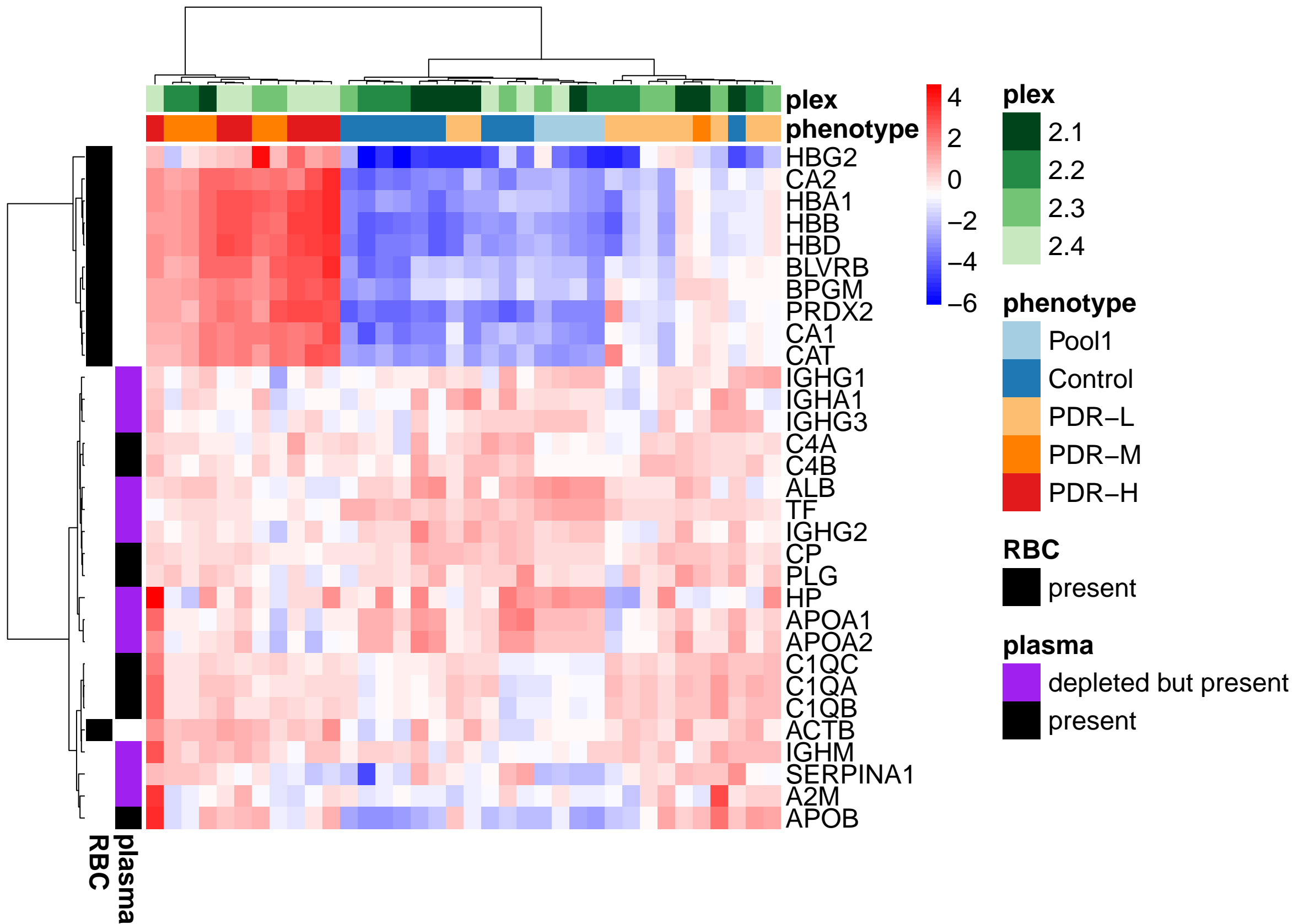

Supplement: Supplementary file 1 — Additional file 1. Supplementary material detailing inputs, protein sets, and analysis results from experiments 1 and 2 can be found here. [file 12014_2021_9328_MOESM1_ESM.zip › Oculomics_tomwgard_CU3-power_analysis-main/outputs/figures/exp2_figures-heatmap_only_erythrocyte_plasma_annotated.pdf]

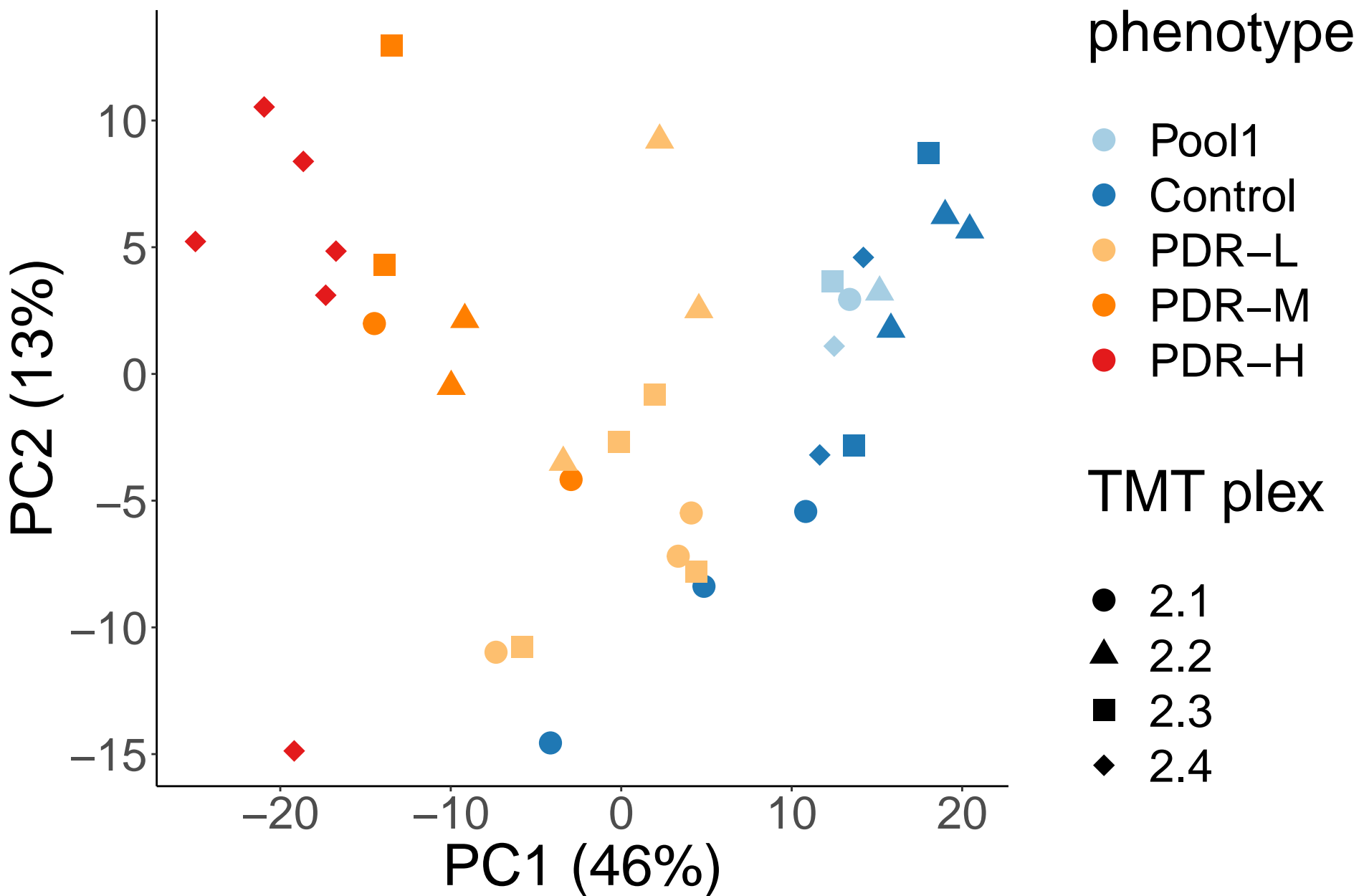

Supplement: Supplementary file 1 — Additional file 1. Supplementary material detailing inputs, protein sets, and analysis results from experiments 1 and 2 can be found here. [file 12014_2021_9328_MOESM1_ESM.zip › Oculomics_tomwgard_CU3-power_analysis-main/outputs/figures/exp2_figures-pca.pdf]

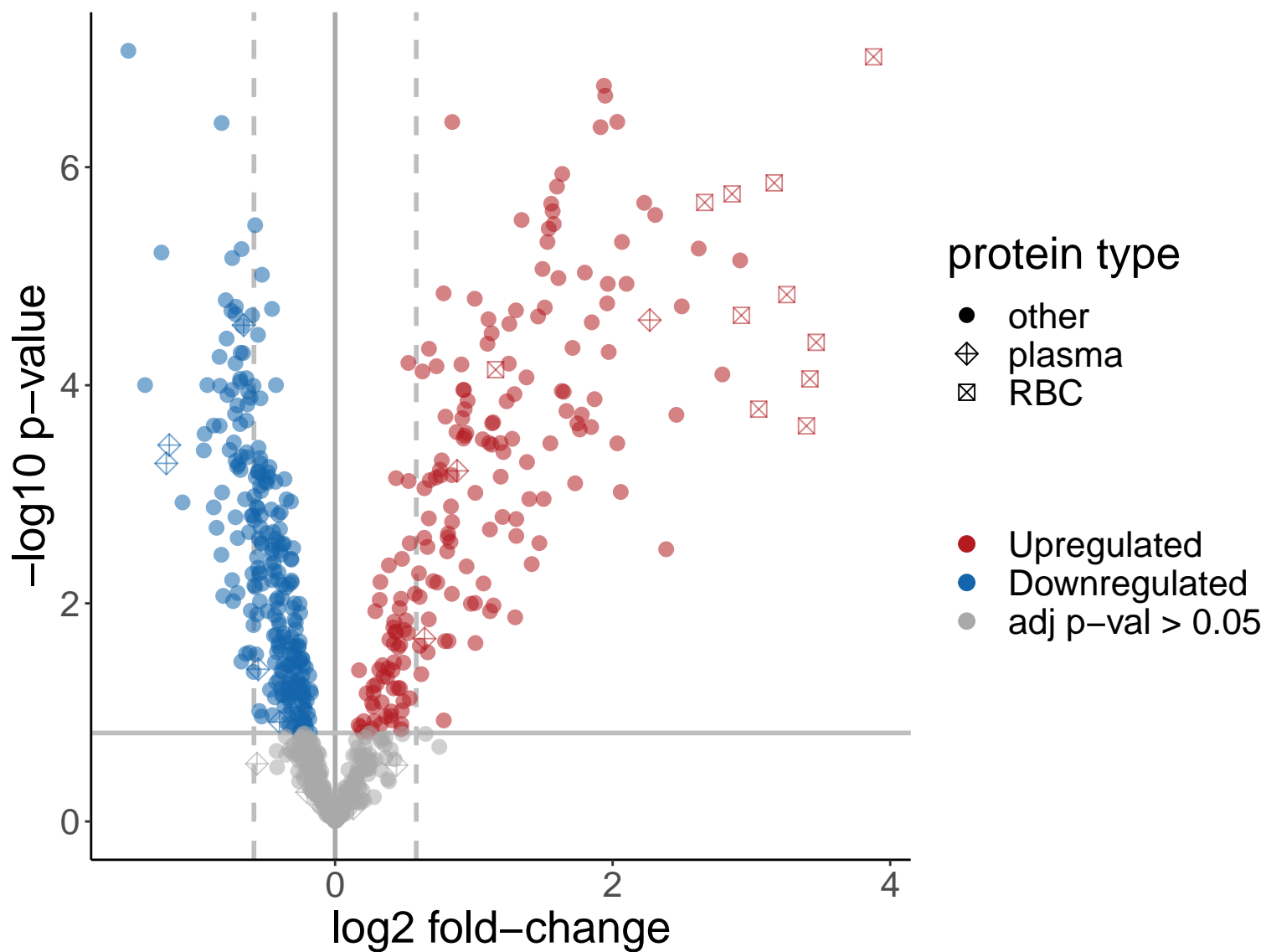

Supplement: Supplementary file 1 — Additional file 1. Supplementary material detailing inputs, protein sets, and analysis results from experiments 1 and 2 can be found here. [file 12014_2021_9328_MOESM1_ESM.zip › Oculomics_tomwgard_CU3-power_analysis-main/outputs/figures/exp2_figures_diffex-volcano.pdf]

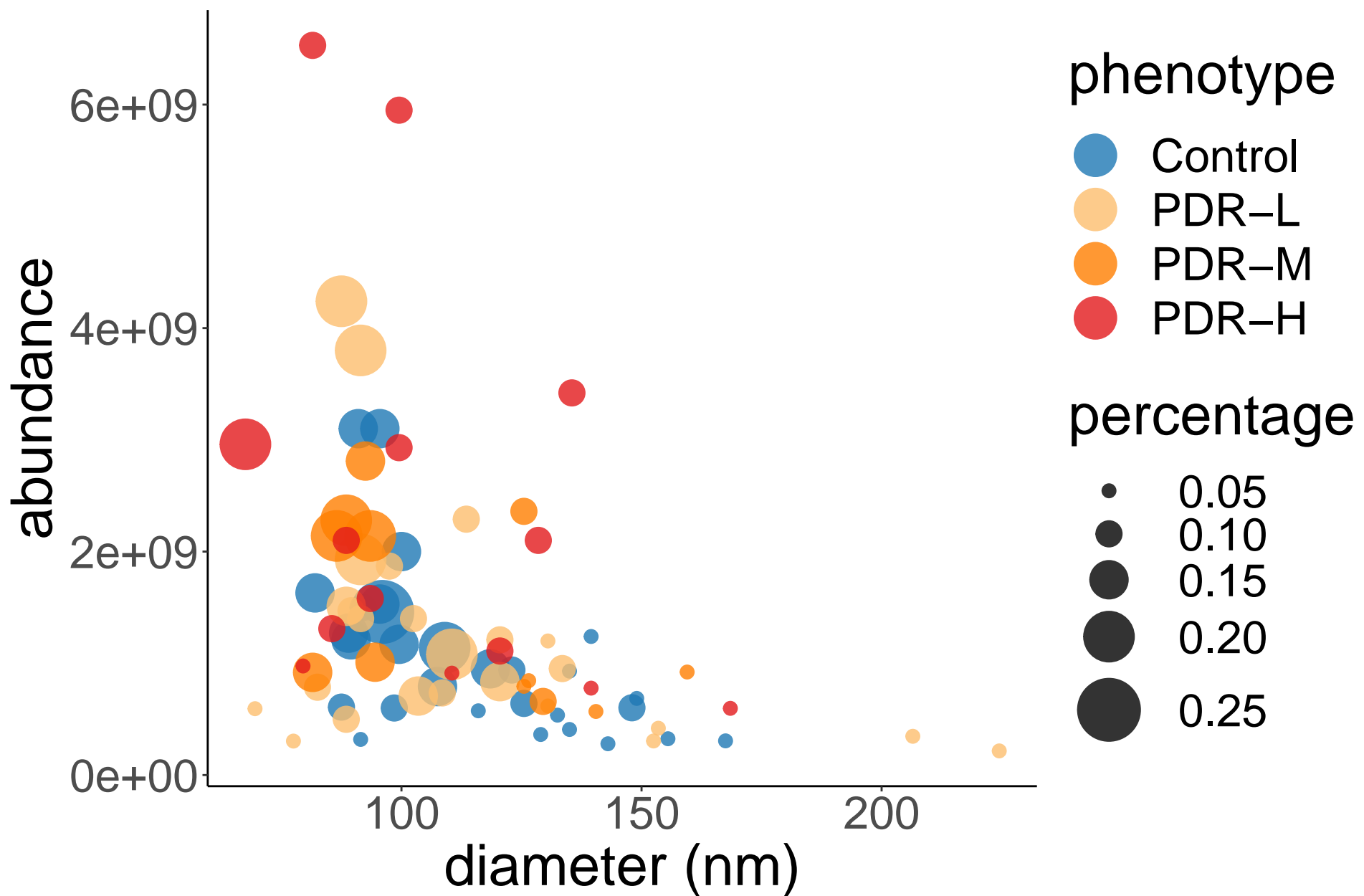

Supplement: Supplementary file 1 — Additional file 1. Supplementary material detailing inputs, protein sets, and analysis results from experiments 1 and 2 can be found here. [file 12014_2021_9328_MOESM1_ESM.zip › Oculomics_tomwgard_CU3-power_analysis-main/outputs/figures/exp2_figures_particle-abundance_x_diameter_scatter.pdf]

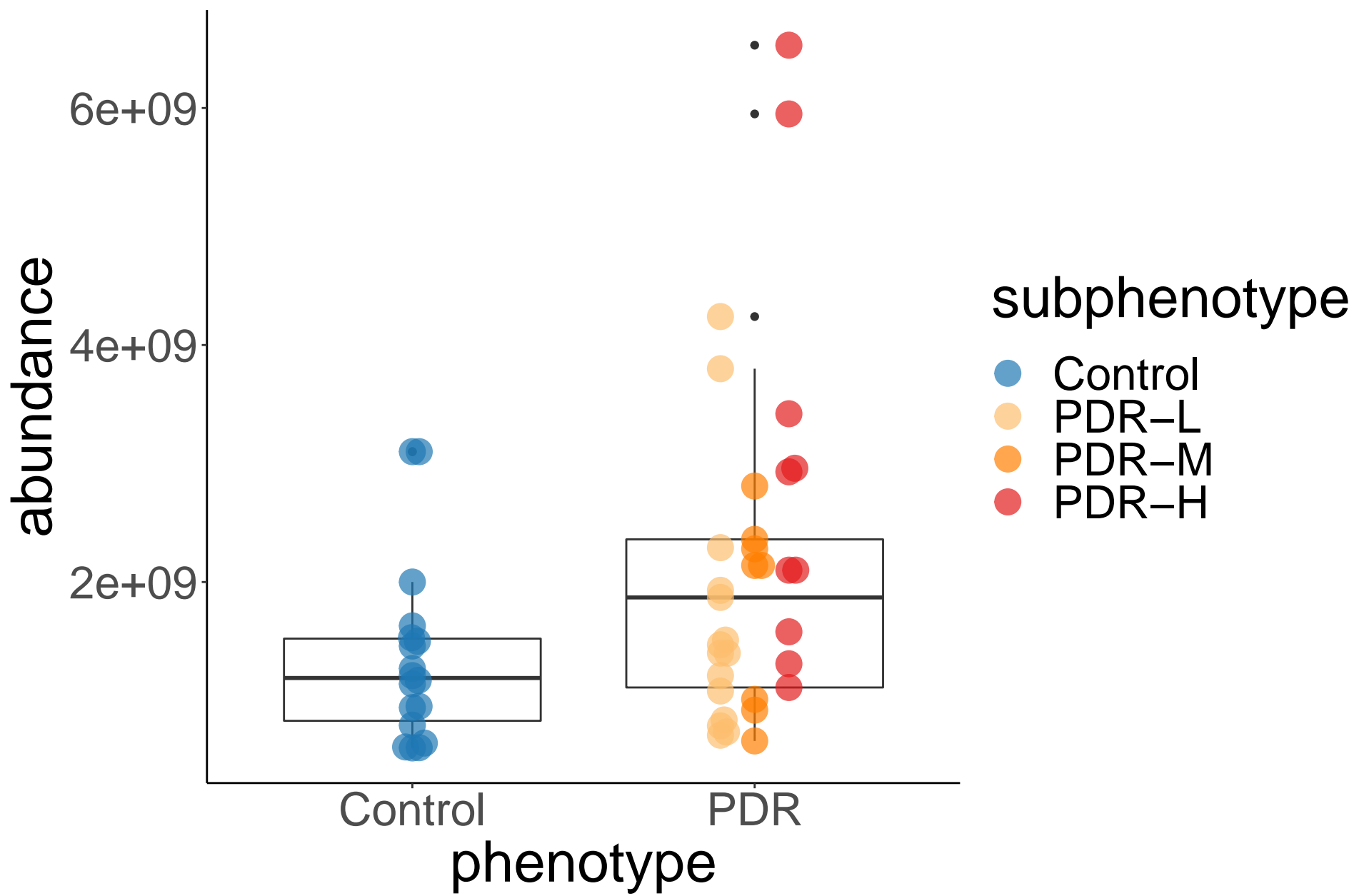

Supplement: Supplementary file 1 — Additional file 1. Supplementary material detailing inputs, protein sets, and analysis results from experiments 1 and 2 can be found here. [file 12014_2021_9328_MOESM1_ESM.zip › Oculomics_tomwgard_CU3-power_analysis-main/outputs/figures/exp2_figures_particle-phenotype_x_abundance.pdf]

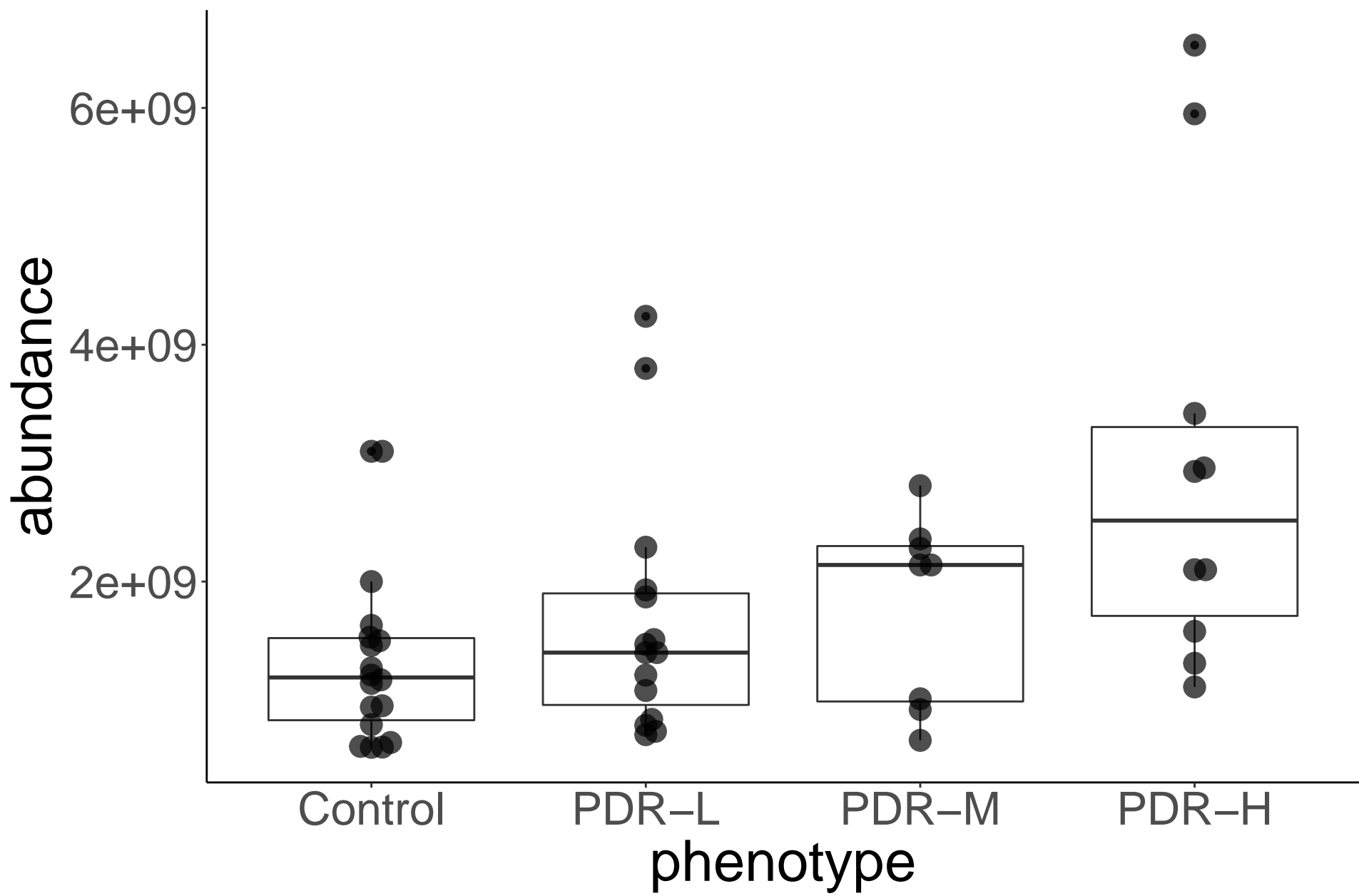

Supplement: Supplementary file 1 — Additional file 1. Supplementary material detailing inputs, protein sets, and analysis results from experiments 1 and 2 can be found here. [file 12014_2021_9328_MOESM1_ESM.zip › Oculomics_tomwgard_CU3-power_analysis-main/outputs/figures/exp2_figures_particle-subphenotype_x_abundance.pdf]

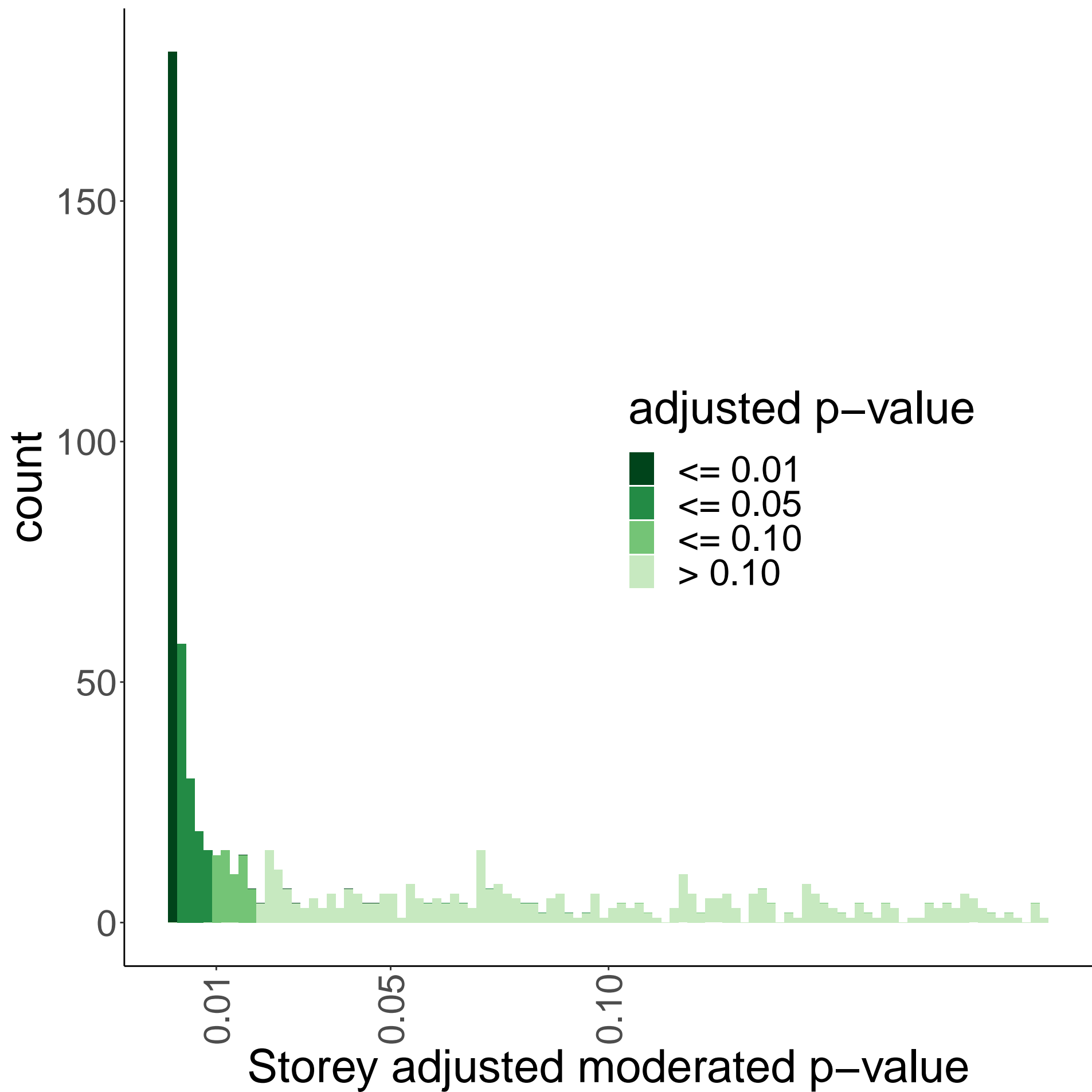

Supplement: Supplementary file 1 — Additional file 1. Supplementary material detailing inputs, protein sets, and analysis results from experiments 1 and 2 can be found here. [file 12014_2021_9328_MOESM1_ESM.zip › Oculomics_tomwgard_CU3-power_analysis-main/outputs/figures/exp2_power-adjusted_pvalue_histogram.pdf]

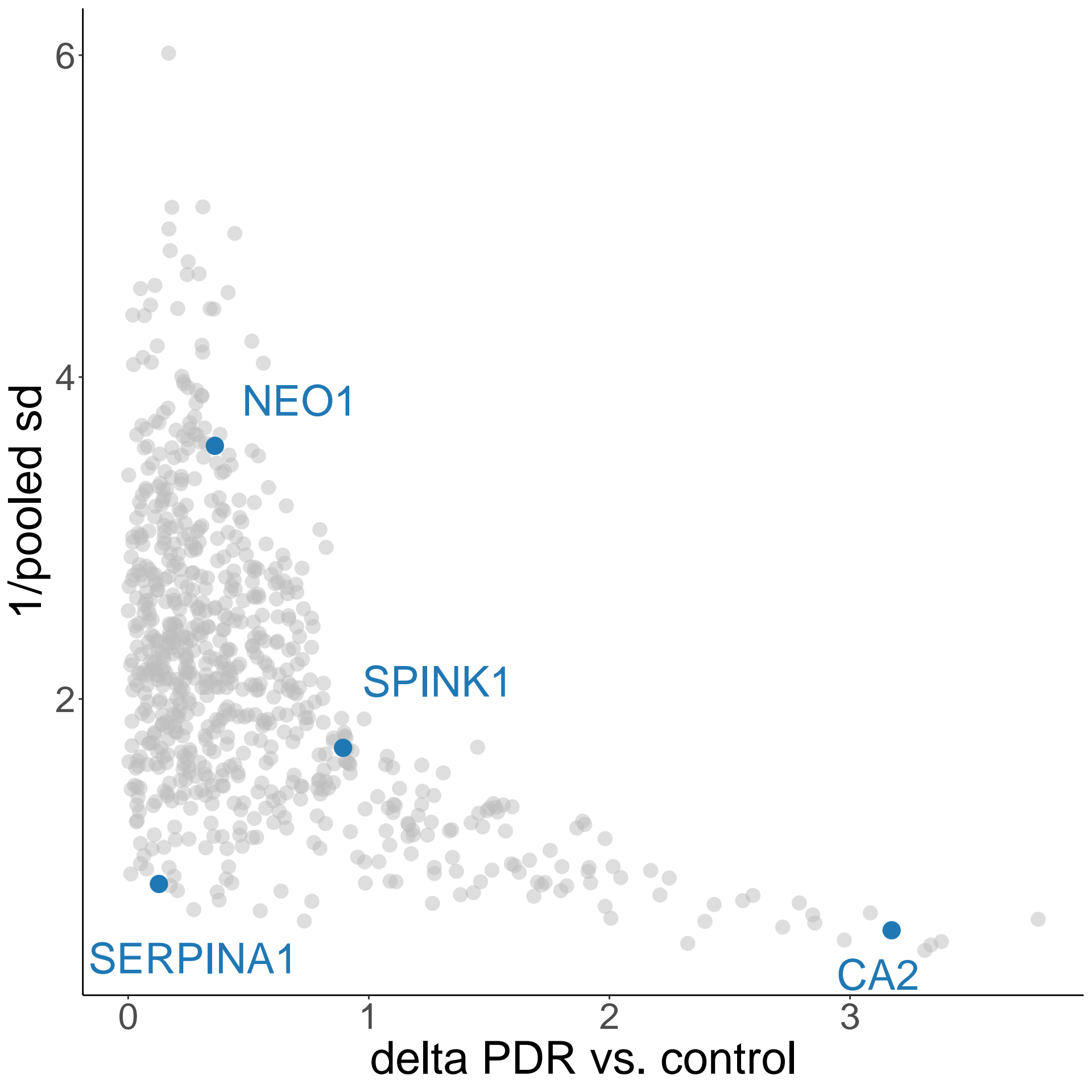

Supplement: Supplementary file 1 — Additional file 1. Supplementary material detailing inputs, protein sets, and analysis results from experiments 1 and 2 can be found here. [file 12014_2021_9328_MOESM1_ESM.zip › Oculomics_tomwgard_CU3-power_analysis-main/outputs/figures/exp2_power-effect_size_scatter.pdf]

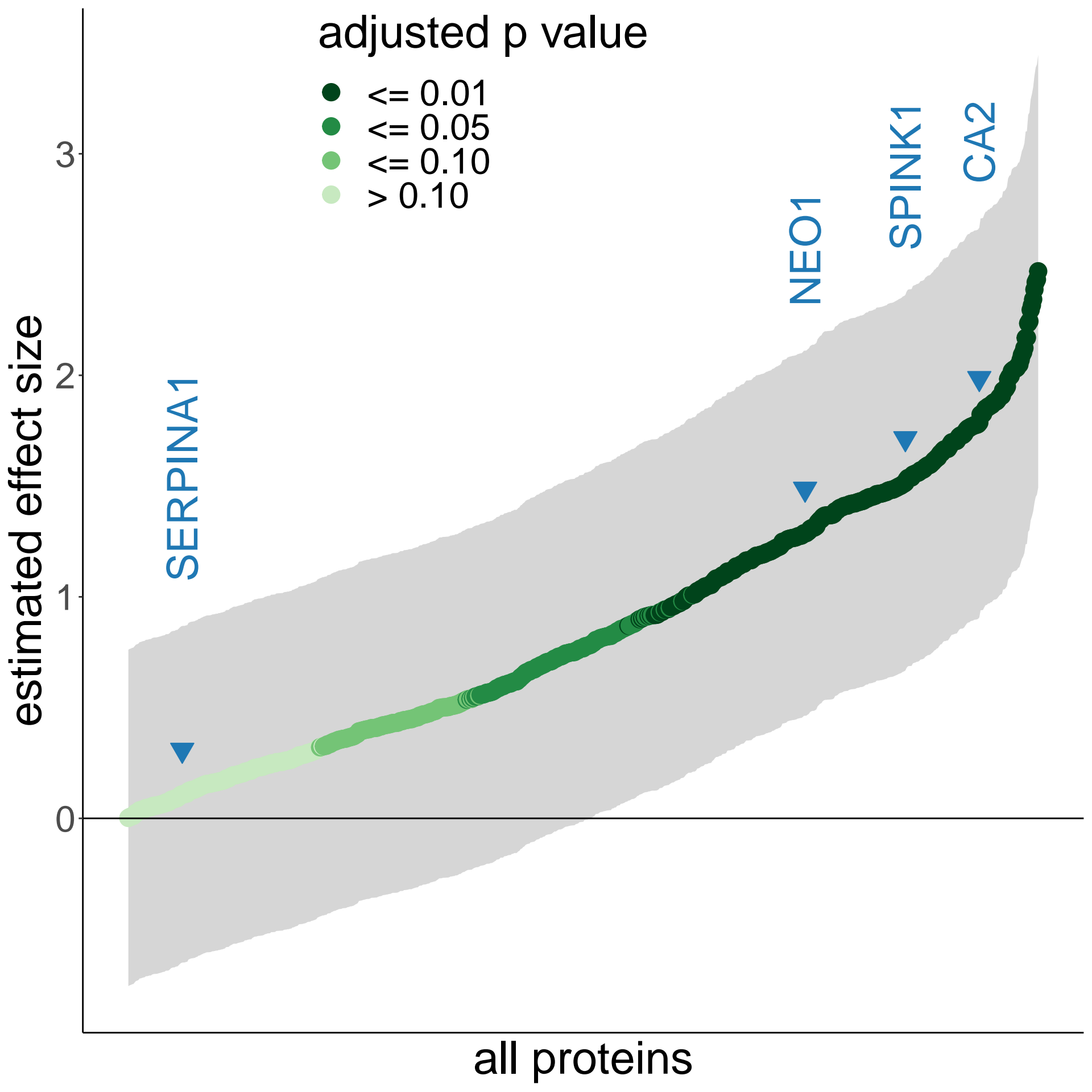

Supplement: Supplementary file 1 — Additional file 1. Supplementary material detailing inputs, protein sets, and analysis results from experiments 1 and 2 can be found here. [file 12014_2021_9328_MOESM1_ESM.zip › Oculomics_tomwgard_CU3-power_analysis-main/outputs/figures/exp2_power-estimated_effect_size_lineplot.pdf]

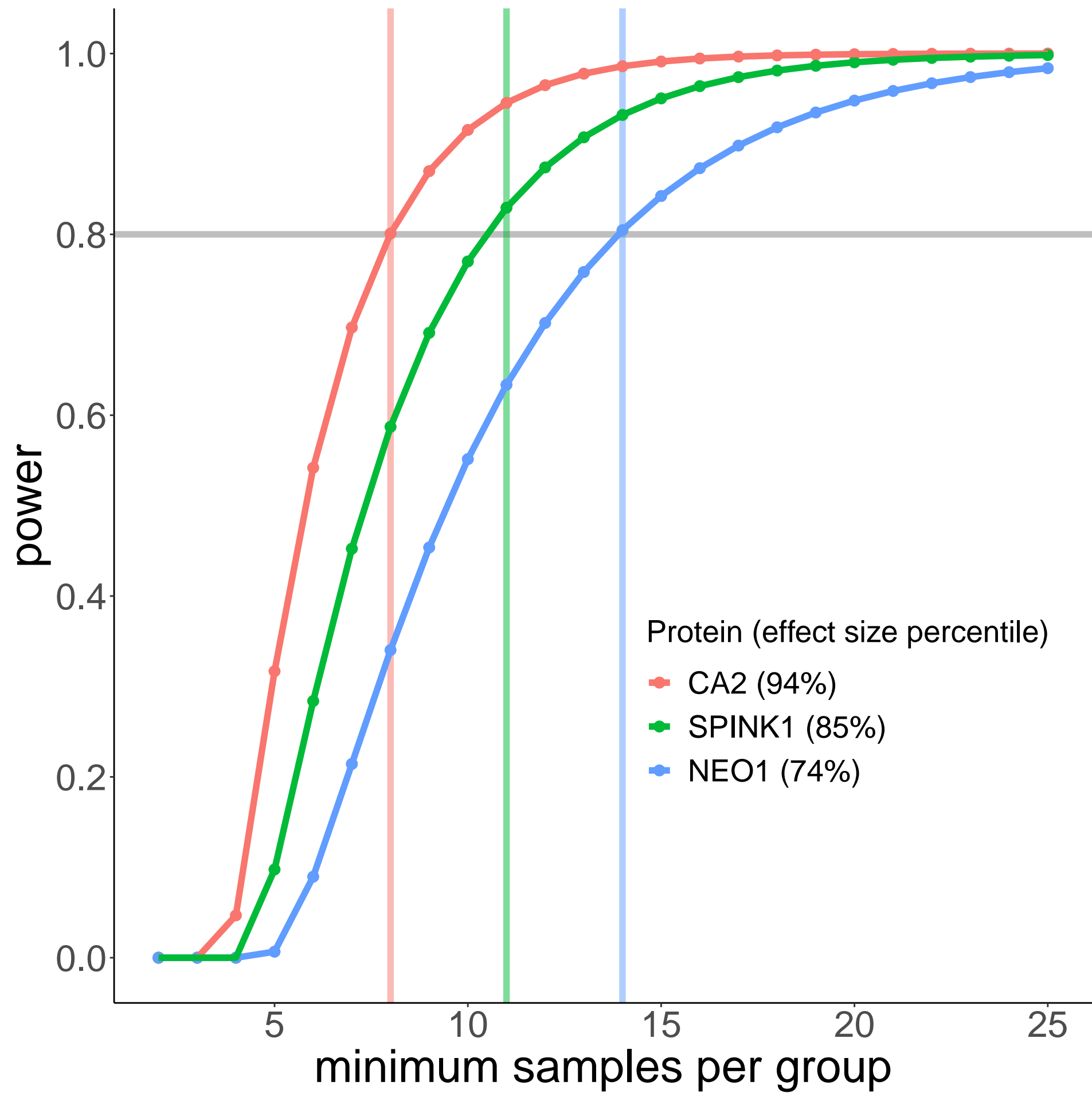

Supplement: Supplementary file 1 — Additional file 1. Supplementary material detailing inputs, protein sets, and analysis results from experiments 1 and 2 can be found here. [file 12014_2021_9328_MOESM1_ESM.zip › Oculomics_tomwgard_CU3-power_analysis-main/outputs/figures/exp2_power-prospective_power_by_effect_size.pdf]

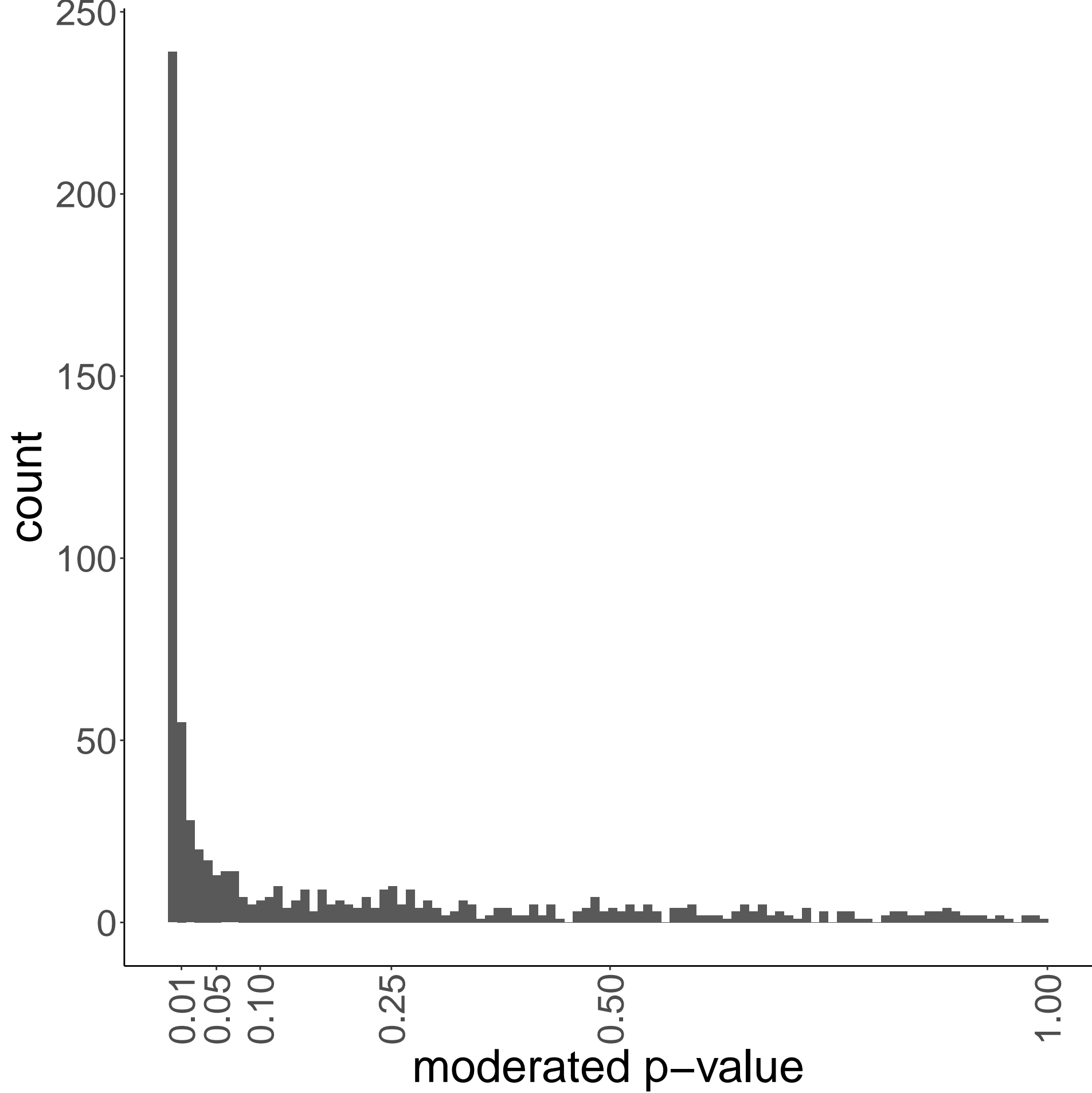

Supplement: Supplementary file 1 — Additional file 1. Supplementary material detailing inputs, protein sets, and analysis results from experiments 1 and 2 can be found here. [file 12014_2021_9328_MOESM1_ESM.zip › Oculomics_tomwgard_CU3-power_analysis-main/outputs/figures/exp2_power-pvalue_histogram.pdf]

normalized protein abundance

SERPINA1

NEO1

SPINK1

CA2

2.5  
0.0  
-2.5  
-5.0

phenotype

PDR Control

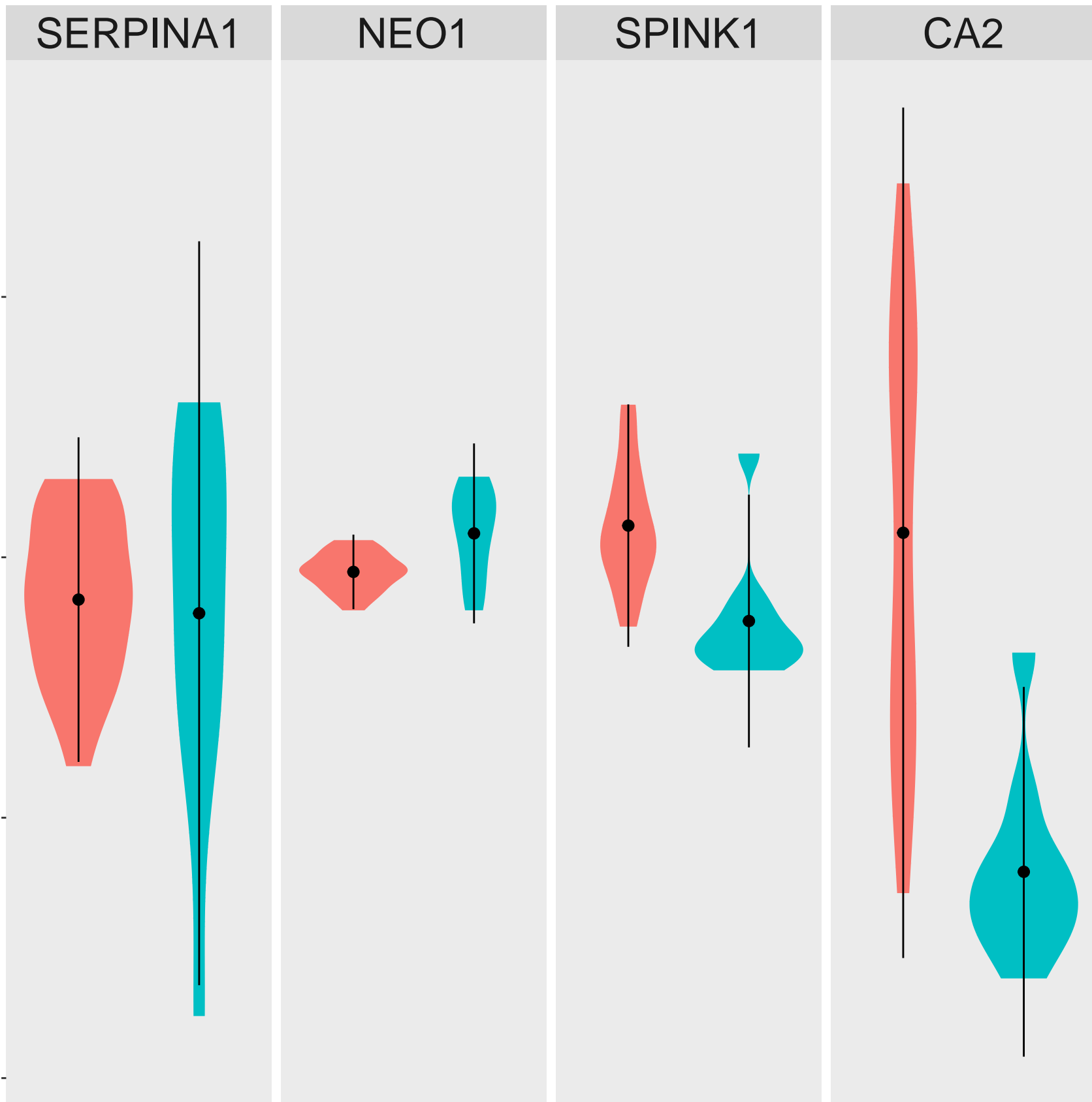

Supplement: Supplementary file 1 — Additional file 1. Supplementary material detailing inputs, protein sets, and analysis results from experiments 1 and 2 can be found here. [file 12014_2021_9328_MOESM1_ESM.zip › Oculomics_tomwgard_CU3-power_analysis-main/outputs/figures/exp2_power-select_genes_phenotype_boxplots.pdf]

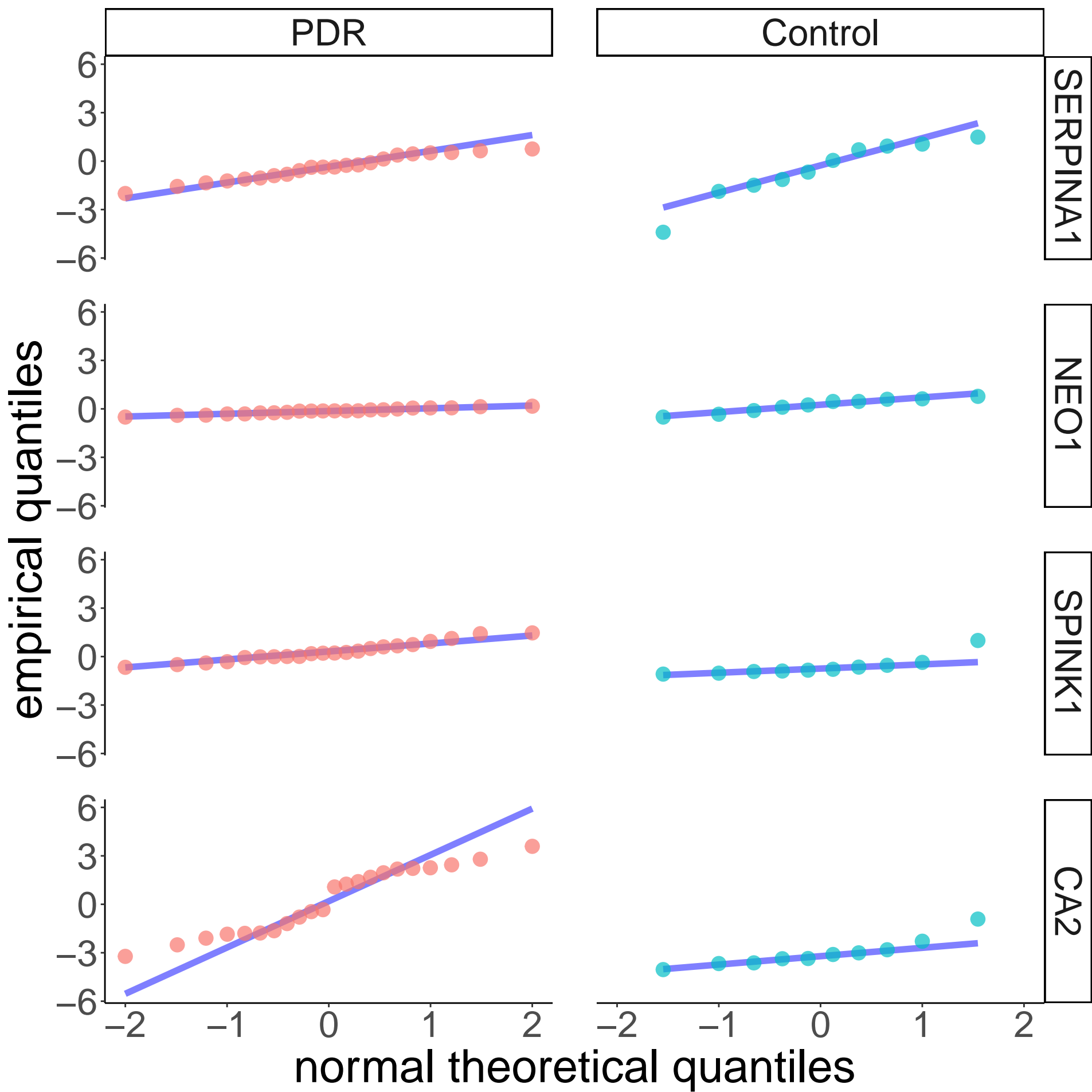

Supplement: Supplementary file 1 — Additional file 1. Supplementary material detailing inputs, protein sets, and analysis results from experiments 1 and 2 can be found here. [file 12014_2021_9328_MOESM1_ESM.zip › Oculomics_tomwgard_CU3-power_analysis-main/outputs/figures/exp2_power-select_genes_qqplots.pdf]

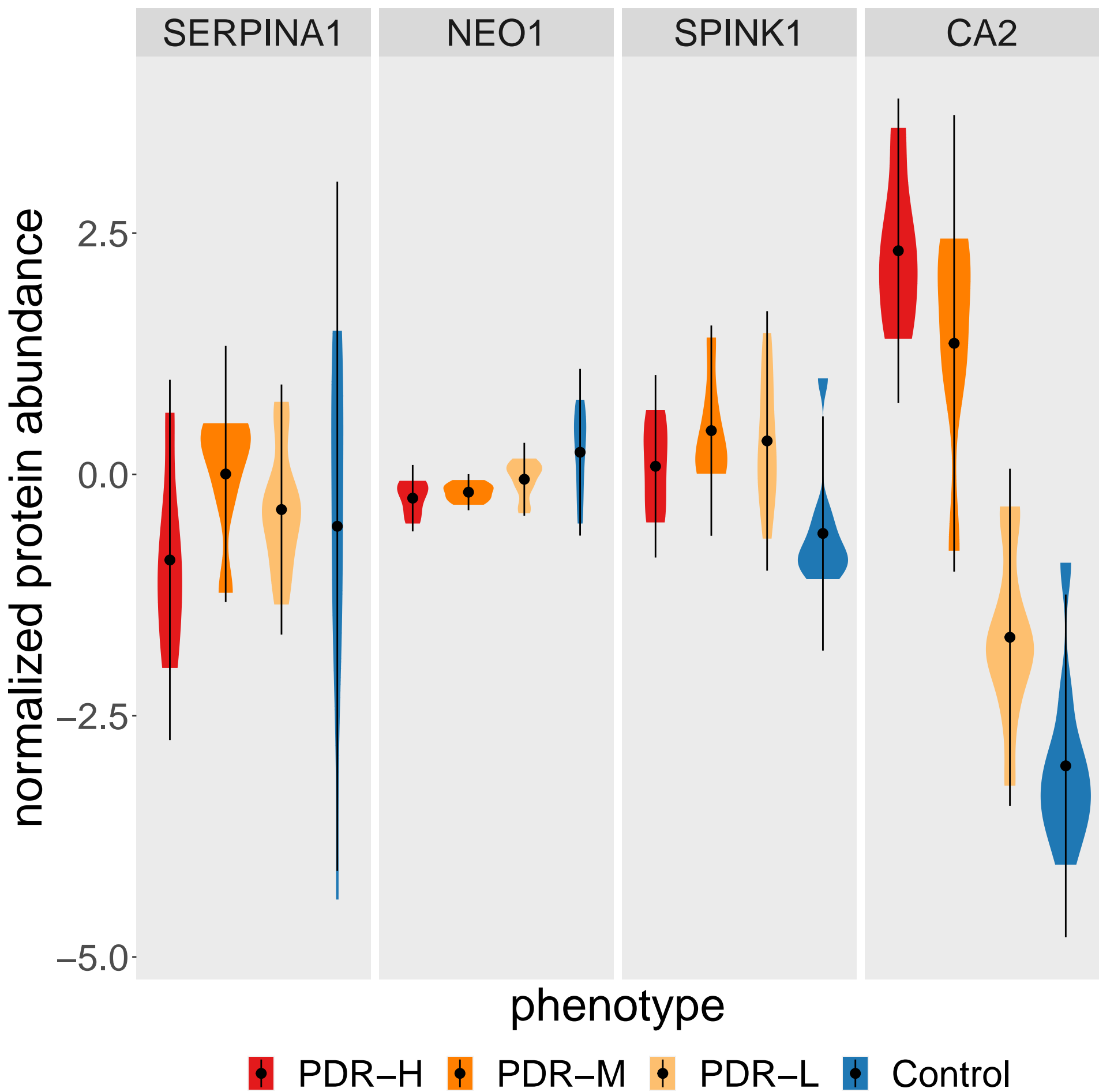

Supplement: Supplementary file 1 — Additional file 1. Supplementary material detailing inputs, protein sets, and analysis results from experiments 1 and 2 can be found here. [file 12014_2021_9328_MOESM1_ESM.zip › Oculomics_tomwgard_CU3-power_analysis-main/outputs/figures/exp2_power-select_genes_subphenotype_boxplots.pdf]

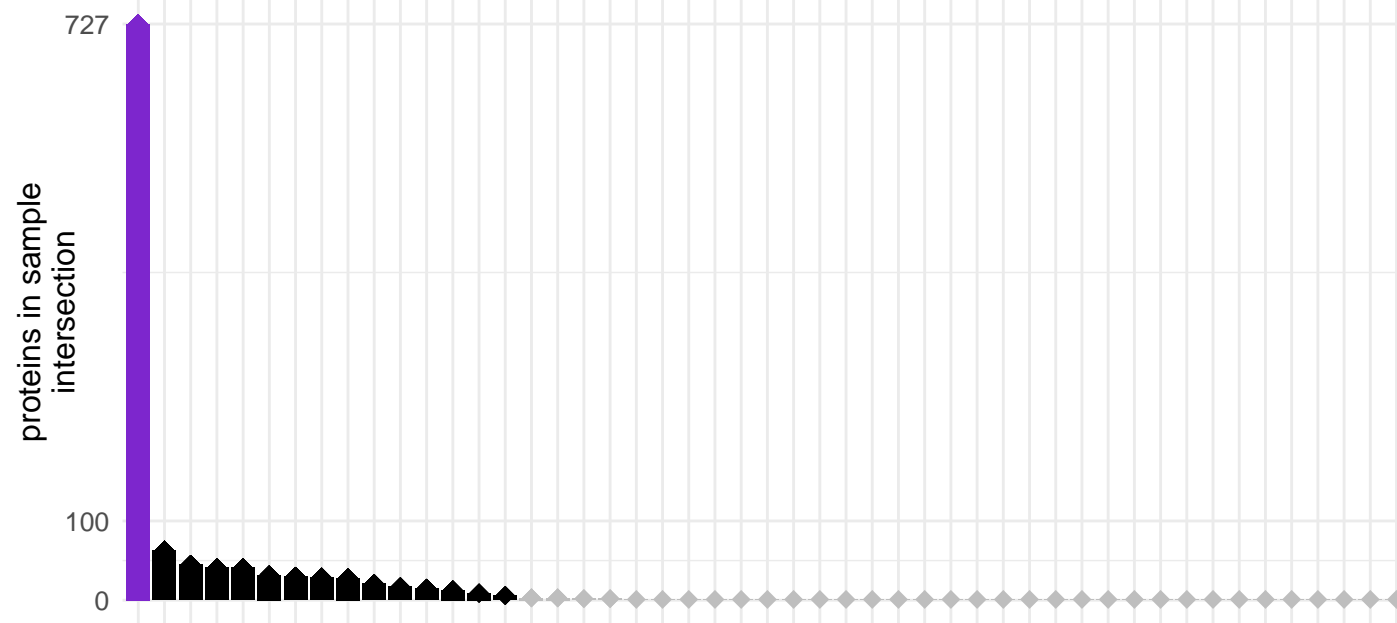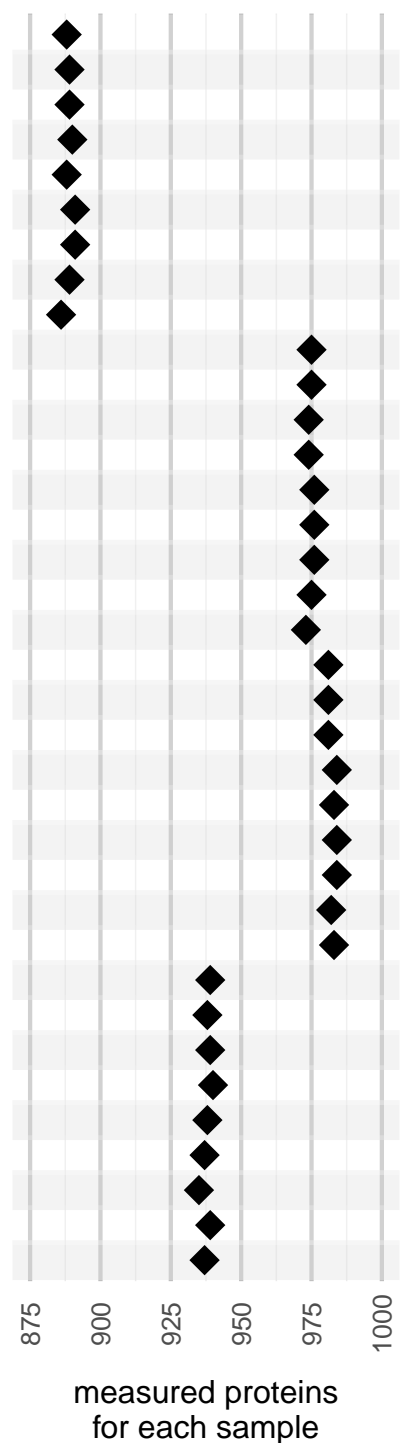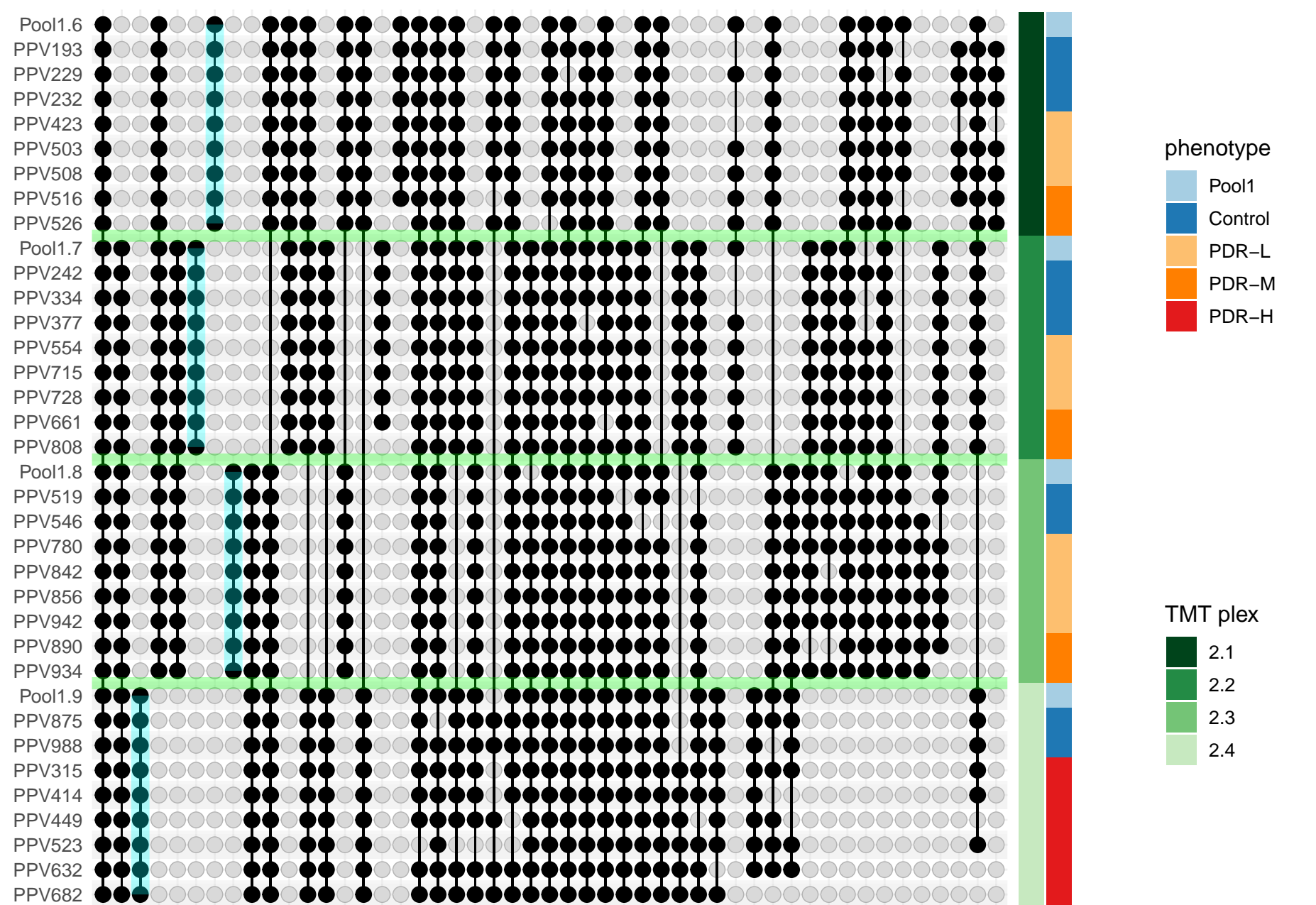

Supplement: Supplementary file 1 — Additional file 1. Supplementary material detailing inputs, protein sets, and analysis results from experiments 1 and 2 can be found here. [file 12014_2021_9328_MOESM1_ESM.zip › Oculomics_tomwgard_CU3-power_analysis-main/outputs/figures/exp2_upset-by_plex.pdf]

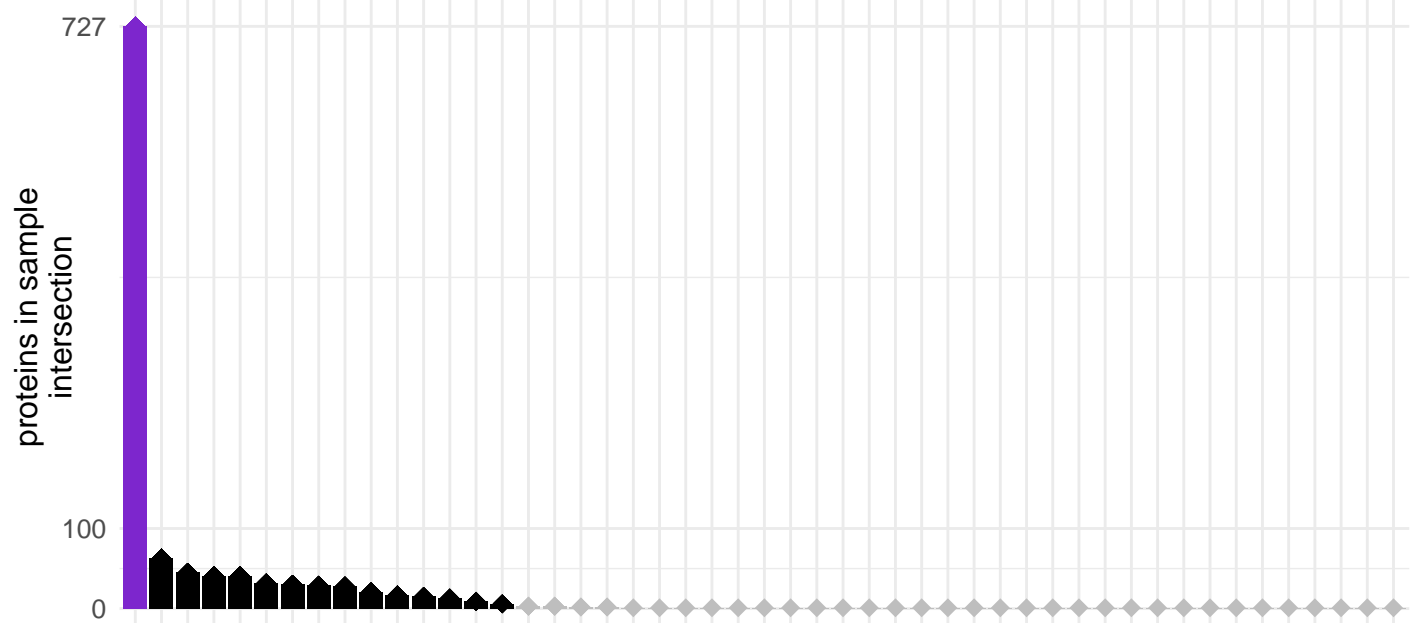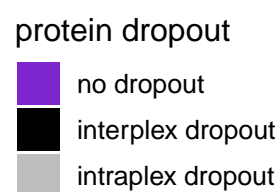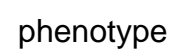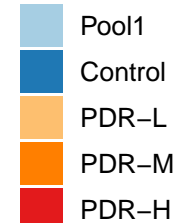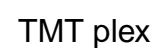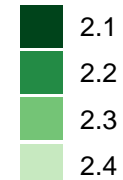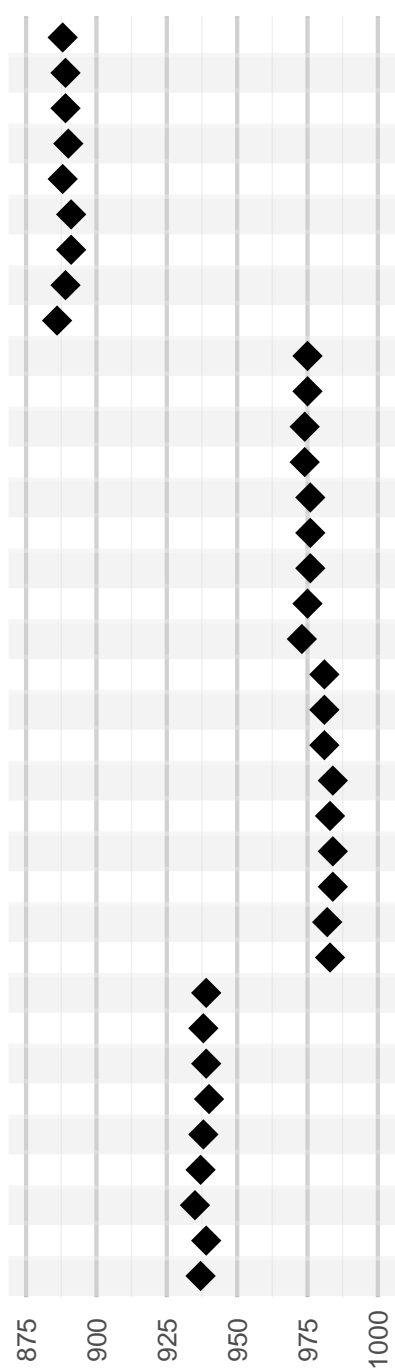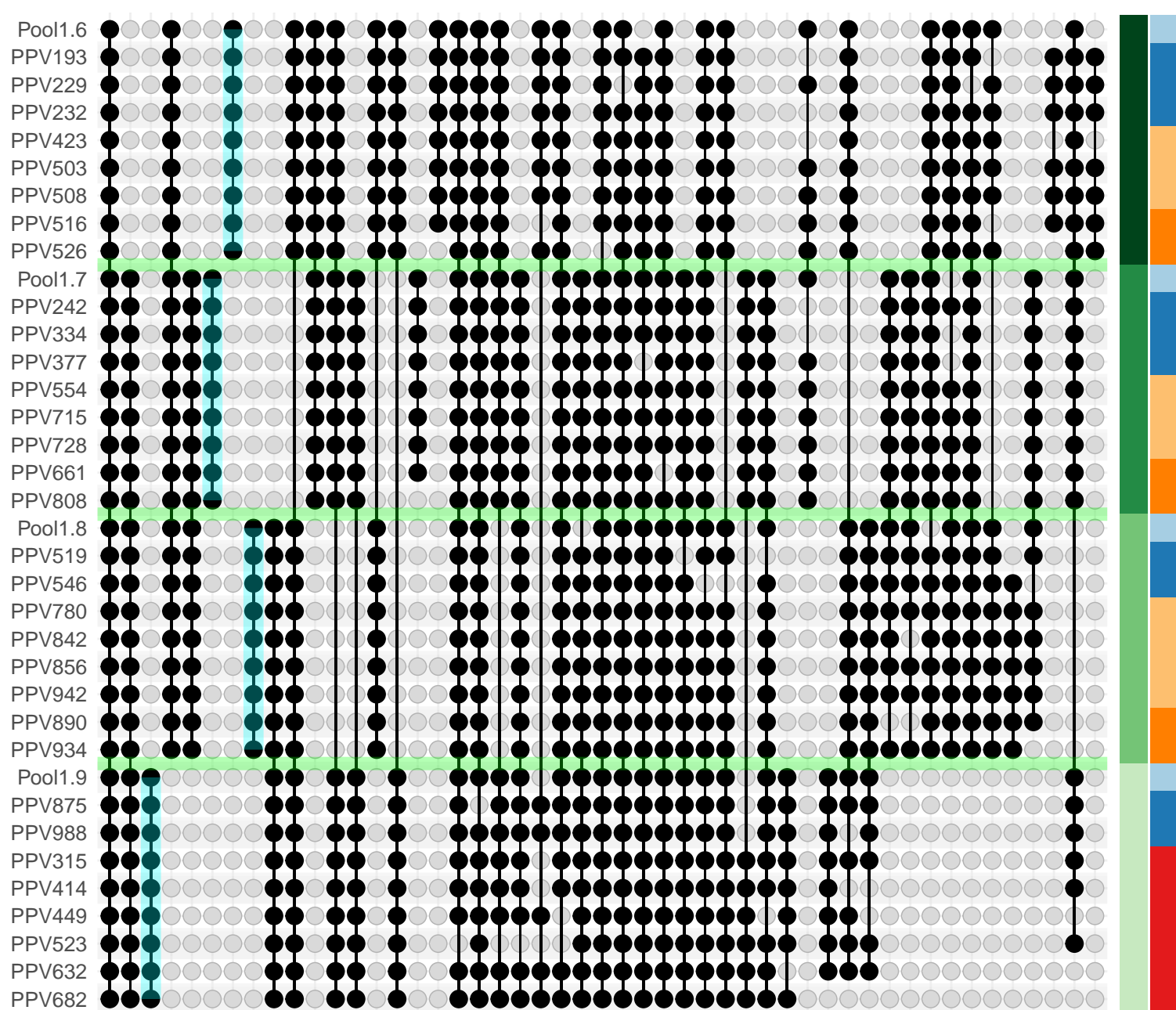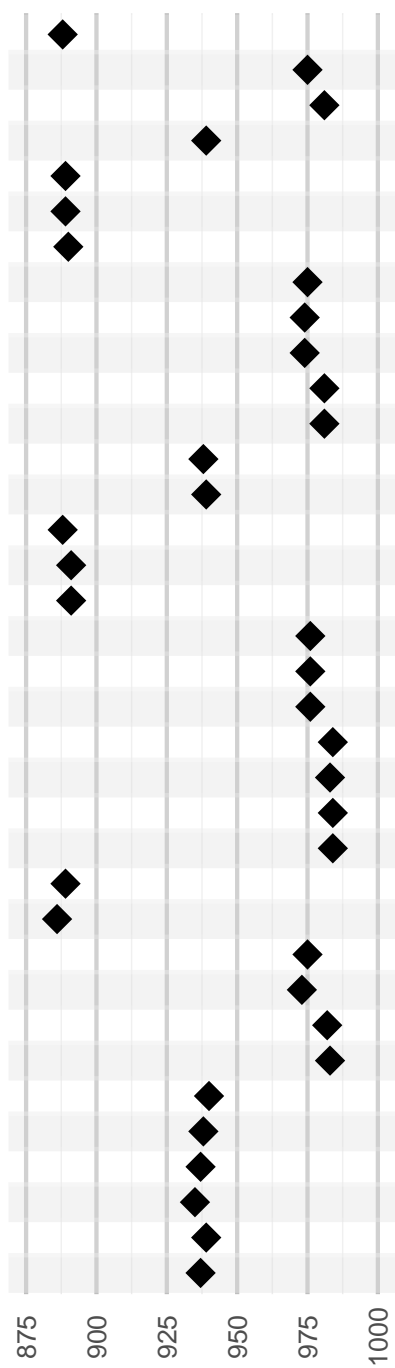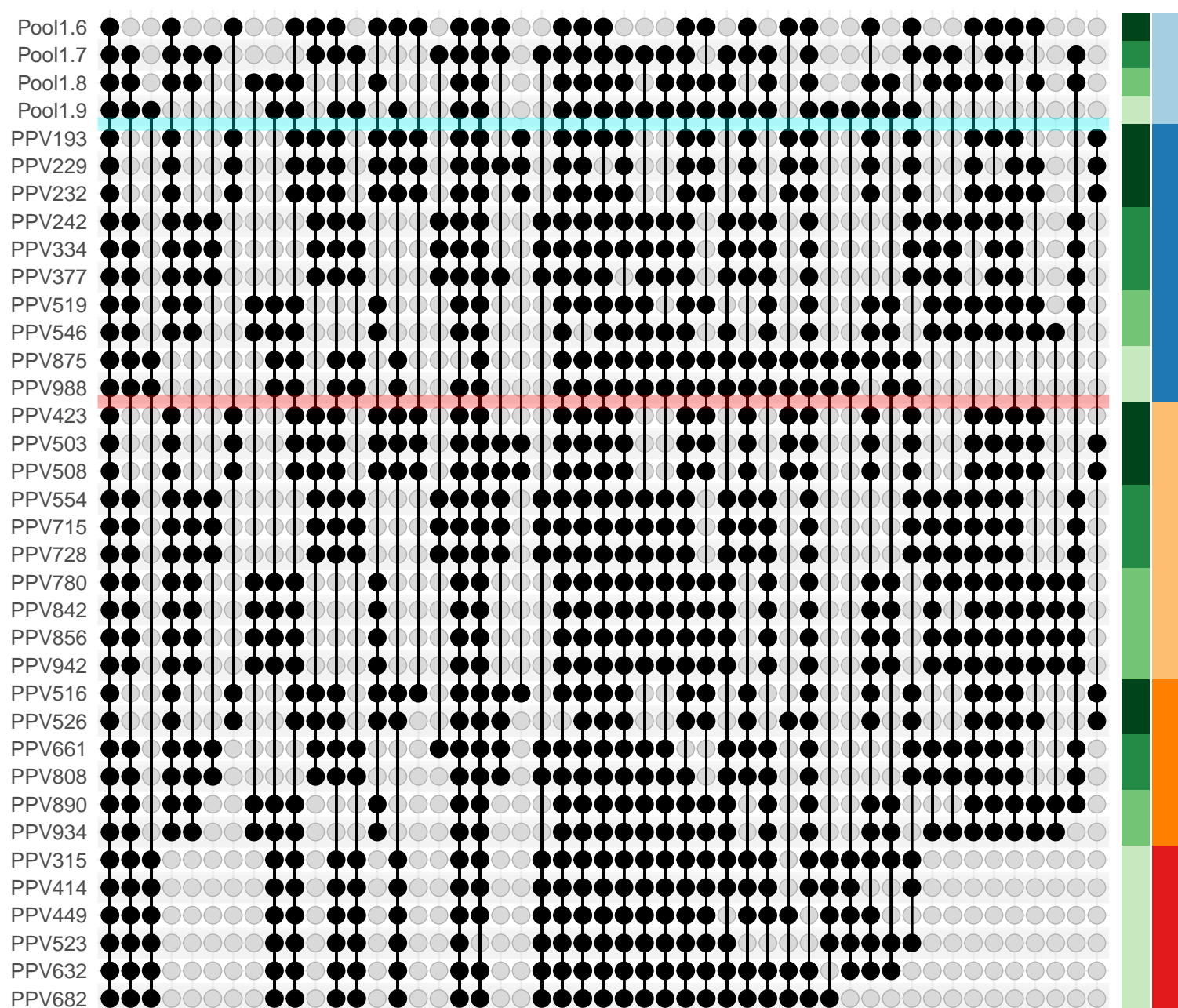

measured proteins  
for each sample

Supplement: Supplementary file 1 — Additional file 1. Supplementary material detailing inputs, protein sets, and analysis results from experiments 1 and 2 can be found here. [file 12014_2021_9328_MOESM1_ESM.zip › Oculomics_tomwgard_CU3-power_analysis-main/outputs/figures/exp2_upset-composite.pdf]
